# *LRRK2* G2019S mutation contributes to mitochondrial transfer dysfunction in a Drp1-STX17-dependent manner

**DOI:** 10.1186/s40035-025-00525-1

**Published:** 2025-12-08

**Authors:** Mei Ding, Fen Wang, Lan-Lan Jiang, Chao Ma, Yu-Wan Qi, Jun-Yi Liu, Juan Li, Mei-Xia Wang, Hong Jin, Jin-Ru Zhang, Cheng-Jie Mao, Xiao-Kang Li, Chun-Feng Liu, Xiao-Yu Cheng

**Affiliations:** 1 Department of Neurology and Clinical Research Center of Neurological Disease, The Second Affiliated Hospital of Soochow University, Suzhou, China; 2 Department of Neurology, Nantong First People’s Hospital, Nantong, China; 3 Jiangsu Key Laboratory of Drug Discovery and Translational Research for Brain Diseases, Institute of Neuroscience, Soochow University, Suzhou, China; 4 Department of Neurology, Xiongan Xuanwu Hospital, Xiongan, China; 5 Department of Neurology, The Fourth Affiliated Hospital of Soochow University, Suzhou, China; 6 School of Pharmacy, Ningxia Medical University, Yinchuan, China; 7 Department of Neurology, Suzhou Municipal Hospital, Suzhou Hospital Affiliated to Nanjing Medical University, Suzhou, China; 8 Laboratory of Transplantation Immunology, National Research Institute for Child Health and Development, Tokyo, Japan

**Keywords:** Parkinson’s disease, *LRRK2* G2019S mutation, Induced pluripotent stem cell, Dopaminergic neuron, Astrocyte, Mitochondrial transfer, Membrane fusion-related protein STX17

## Abstract

**Background:**

Previous studies have shown that astrocytes can transfer healthy mitochondria to dopaminergic (DA) neurons, which may serve as an intrinsic neuroprotective mechanism in Parkinson’s disease (PD). *LRRK2* G2019S is the most common pathogenic mutation associated with PD. In this study, we explored whether mitochondrial transfer is influenced by genetic and environmental factors and whether dysfunction in this process is one of the mechanisms of the pathogenic *LRRK2* G2019S mutation.

**Methods:**

DA neurons and astrocytes were differentiated from induced pluripotent stem cells generated from the peripheral blood of a healthy individual and a PD patient carrying the *LRRK2* G2019S mutation. A coculture system of astrocytes and DA neurons was established to explore the pathogenic mechanisms of *LRRK2* G2019S.

**Results:**

Exposure to the environmental toxin rotenone impaired mitochondrial transfer from astrocytes to DA neurons. Compared with the co-culture system from the healthy participant, the co-culture system harboring the *LRRK2* G2019S mutation experienced more pronounced damage. Specifically, STX17 was colocalized with the mitochondrial outer membrane marker TOM20, and its knockdown caused damage to mitochondrial transfer. Drp1 interacted with STX17. *LRRK2* G2019S-mutant astrocytes exhibited markedly increased phosphorylation of Drp1 at Ser616 upon rotenone exposure. Moreover, the degree of colocalization of STX17 with TOM20 decreased. The Drp1 phosphorylation inhibitor DUSP6 restored the colocalization of STX17 and TOM20, as well as the mitochondrial transfer efficiency and neuronal survival.

**Conclusions:**

The impairment of mitochondrial transfer is a potential pathogenic mechanism associated with *LRRK2* G2019S mutation. The molecular mechanisms of mitochondrial transfer were observed to occur through a Drp1-STX17-dependent pathway. Notably, inhibitors for Drp1 Ser616 phosphorylation may offer neuroprotection through mitigating mitochondrial transfer impairments. This study provides novel insights into the pathogenesis of PD and the development of new therapeutic targets.

**Graphical abstract:**

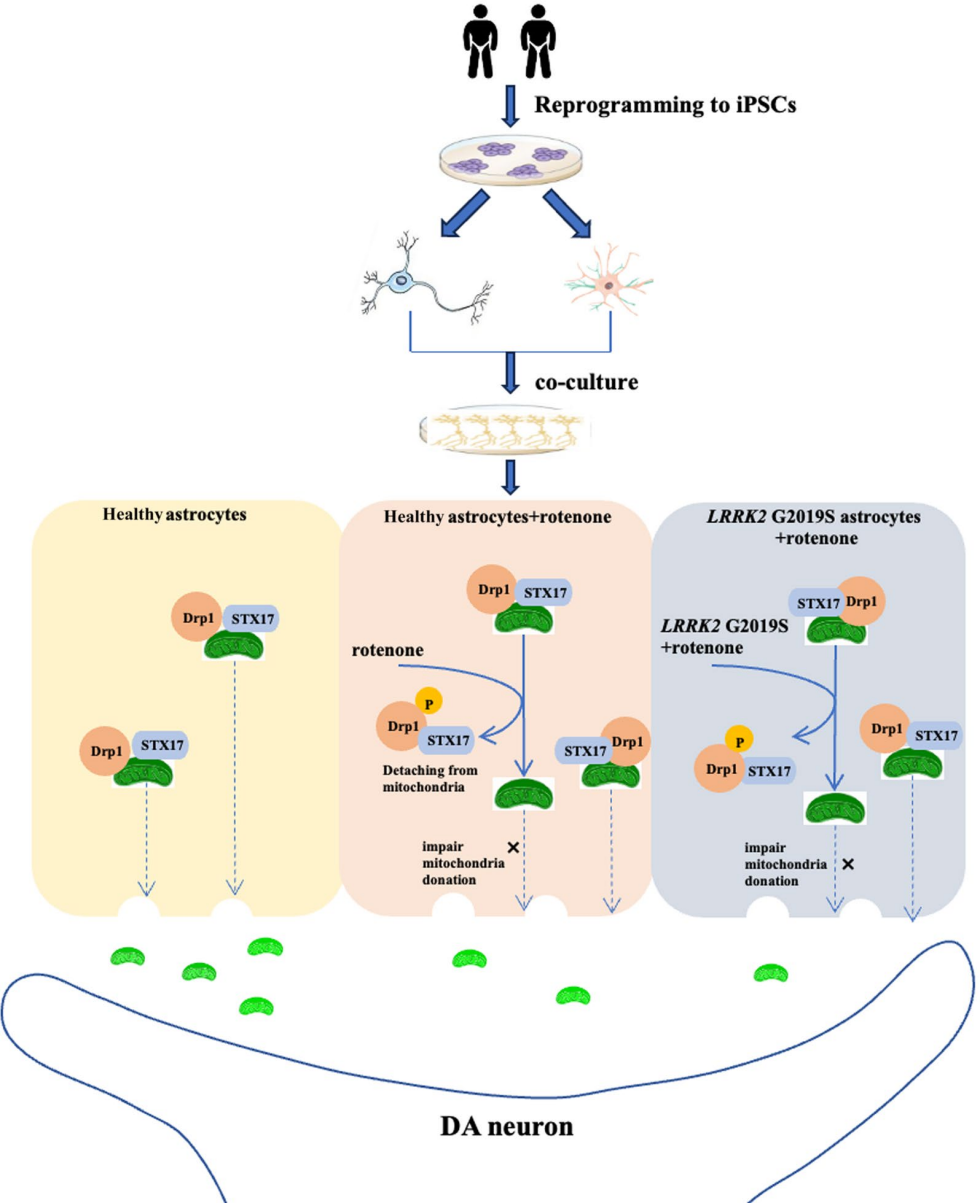

**Supplementary Information:**

The online version contains supplementary material available at 10.1186/s40035-025-00525-1.

## Background

Parkinson’s disease (PD) is the second most prevalent neurodegenerative disorder after Alzheimer’s disease [1] and is characterized by degeneration of dopaminergic (DA) neurons in the substantia nigra pars compacta. Currently, dopamine replacement therapy remains the primary treatment; however, this therapeutic approach is unable to cure the disease or delay its progression. Therefore, investigating potential neuroprotective mechanisms that inhibit neuronal apoptosis is a promising research direction.

While aging, genetic and environmental factors contribute to PD development, the precise pathogenesis remains unclear. Mutations in the leucine-rich repeat kinase 2 (*LRRK2*) gene are among the most significant genetic risk factors for PD and are responsible for approximately 10% of familial cases and 3.6% of sporadic cases [2, 3]. *LRRK2* mutations intensify the effects of environmental toxins, jointly promoting disease development. LRRK2 is a protein kinase predominantly localized in the cytoplasm and mitochondrial outer membrane and influences cellular functions by interacting with target proteins through its various domains [4]. The LRRK2 catalytic core comprises a leucine-rich repeat domain, a GTPase-active Ras Of Complex (ROC) domain, C-terminal of ROC involved in protein dimerization, and a WD40 domain [5]. The *LRRK2* G2019S mutation is a well-established pathogenic variant associated with PD [6]. Current research indicates a close association between the *LRRK2* G2019S mutation and mitochondrial abnormalities [7–9]. Located on the mitochondrial outer membrane, the LRRK2 protein affects several mitochondrial physiological processes, including mitophagy, mitochondrial fusion, fission, and transport [10, 11]. Neuropathological, genetic, and biochemical studies have revealed that mitochondrial dysfunction is a key aspect of PD pathogenesis [12]. Thus, *LRRK2* mutations likely contribute to PD pathogenesis by disrupting mitochondrial functions. The pathological *LRRK2* G2019S mutation markedly increases the LRRK2 kinase activity, contributing to various pathological processes, such as mitophagy disruption, mitochondrial DNA damage, and abnormal mitochondrial fission [13]. Our previous studies have demonstrated that astrocytes can transfer healthy mitochondria to DA neurons, thereby rescuing damaged neurons in PD models [14]. “Mitochondrial transfer” as an essential mechanism for mitochondrial replenishment and repair, has been reported across various disease models [15–17]. However, whether the *LRRK2* G2019S mutation causes impairment of mitochondrial transfer-mediated neural repair remains to be elucidated.

Syntaxin 17 (STX17) is a crucial mitochondrial protein located on the mitochondrial outer membrane via transmembrane domains and is integral to various mitochondrial physiological processes [18]. Recent studies have highlighted the involvement of STX17 in regulating mitophagy, mitochondria‒endoplasmic reticulum crosstalk, and other essential activities, primarily through its role in mediating membrane fusion [19, 20]. We previously observed that STX17 is important for mitochondrial transfer. Notably, STX17 is among the first identified soluble *N*-ethylmaleimide-sensitive factor attachment protein receptor (SNARE) proteins. The SNARE complex mediates membrane fusion and plays a central role in the secretion of substances or structures such as neurotransmitters, lysosomes, and exosomes [21, 22]. The SNARE proteins are categorized into three groups: syntaxins (STXs), synaptosomal-associated proteins (SNAPs), and vesicle-associated membrane proteins (VAMPs), which together form the SNARE complex, mediating most membrane fusion events, including exocytosis [23]. The question of whether STX17 mediates the exocytosis of mitochondria by astrocytes through SNARE remains unclear. Recent research on the localization of STX17 within intracellular subregions has indicated that its dynamic positioning is closely tied to its functional state. Specifically, STX17 in the endoplasmic reticulum, mitochondrial outer membrane, mitochondrial-associated membrane system, and cytoplasm mediates distinct physiological processes [22]. Dynamin-related protein 1 (Drp1), which is located in the mitochondrial outer membrane, is a mitochondrial fission factor and its function is regulated by phosphorylation [24]. In particular, LRRK2 regulates mitochondrial dynamics by phosphorylating Drp1 at Ser616 [25]. Furthermore, hunger signals modulate the interaction between STX17 and Drp1, leading to the shedding of STX17 from mitochondria [21]. These findings raise the hypothesis that LRRK2, through Drp1 phosphorylation, can regulate STX17 localization and thereby influence mitochondrial export processes.

In this study, we used the induced pluripotent stem cell (iPSC) technology to generate human DA neurons and astrocytes with specific genetic backgrounds to establish an “astrocyte-DA neuron” coculture system. We first investigated the mitochondrial transfer efficiency in the *LRRK2* G2019S background and then explored how the pathological LRRK2 G2019S protein induces mitochondrial export dysfunction through the Drp1-STX17 axis. Finally, we evaluated the reparative effect of the Drp1 phosphorylation inhibitor DUSP6 on mitochondrial transfer disorders, as well as its protective effect on neurons. The aim of this study was to reveal a novel mechanism through which *LRRK2* G2019S contributes to PD pathogenesis and provide a new target for PD treatment.

## Materials and methods

### Generation and passaging of human-induced pluripotent stem cells (hiPSCs)

Human blood samples were collected in sodium citrate tubes from a healthy donor and a PD patient with *LRRK2* G2019S mutation (NM_198578; exon 41; c.6055G > A chr12-40,734,202; p. G2019S), as approved by the Second Affiliated Hospital of Soochow University Clinical Trial Laboratory Service and the Ethics Committees. Informed consent was obtained from both participants. The *LRRK2* G2019S mutation was verified by Sanger sequencing. The PD patient was diagnosed by a professional neurologist on the basis of the International Parkinson and Movement Disorder Society diagnostic criteria (2015 edition). The demographic characteristics are provided in Fig. S1. Peripheral blood mononuclear cells (PBMCs) were isolated by gradient centrifugation. iPSCs were generated using an iPSC Reprogramming Kit (CytoTune-iPS 2.0 Sendai Reprogramming Kit, Thermo Fisher Scientific, A34546, Waltham, MA) containing Sendai virus vectors as previously described [26, 27]. The generated iPSCs were confirmed to have normal karyotypes (Fig. S2a, b) and were free from mycoplasma contamination (Fig. S2e). The hiPSCs were cultured under feeder-free conditions in Essential 8 Basal Medium (Thermo Fisher Scientific, A1517001). The medium was changed daily, and the cells were passaged every 5–7 days, depending on plate confluence. During passaging, the cells were washed with PBS and incubated with a dispersing enzyme (Invitrogen, 17105041, Waltham, MA) for 5 min. During iPSC culture and the initial differentiation, typical hiPSC colonies were screened by light microscopy (Fig. S2c, d) [28, 29]. The colonies were subsequently mechanically dissociated and plated onto Matrigel-coated plates.

### DA neuron differentiation

Generation of rosette neural stem cells (NSCs) with DA potential was facilitated with dual SMAD inhibition by Noggin and SB431542. Specifically, the iPSCs were incubated for 4 days in neural induction medium, consisting of DMEM/F12 (Gibco, 11320033, Waltham, MA), 1 × NEAA (Gibco, 11140050), 1 × sodium pyruvate (Gibco, 11360070), 1 × GlutaMax (Gibco, 35050061), 1 × 2-mercaptoethanol (Gibco, 21985023), and 1 × penicillin‒streptomycin (Gibco, 15140122), supplemented with 500 ng/mL Noggin (R&D Systems, 3344-NG-100, Minneapolis, MN) and 10 µM SB431542 (TargetMol, T1726, Boston, MA). Afterward, the cells were cultured for an additional 5 days in the DA neuron induction medium supplemented with 500 ng/ml Noggin (R&D Systems, 3344-NG-100), 200 ng/mL Sonic hedgehog (SHH) (R&D Systems, 1845-S), and 1 × N-2 (Thermos, 17502048). Subsequently they were further incubated for 5 days in DA neuron induction medium including brain-derived neurotrophic factor (BDNF, 20 ng/mL; Thermo, 450–02-10UG), SHH (200 ng/mL; R&D Systems, 1845-S), *L*-ascorbic acid (L-AA, 200 µM; Sigma, A4544, Darmstadt, Germany), and fibroblast growth factor 8b (100 ng/mL; Thermo, 100–25-100). The medium was changed every other day. To further mature the DA neurons, the cells were exposed to N2 medium supplemented with BDNF (20 ng/mL, Thermo, 450–02-10UG), glial-derived neurotrophic factor (GDNF, 20 ng/mL, Thermo, 450–10-10), transforming growth factor β3 (1 µM; Thermos, 100-36E-10), L-AA (200 µmol/L, Sigma, A4544), and cAMP (1 mM; Sigma, D0260-100MG) for an additional 14 days. Mature DA neurons exhibited a typical morphology, and immunocytochemical staining for neuron-specific class III β-tubulin (Tuj1) and tyrosine hydroxylase (TH) was used to confirm their identity.

### Astrocyte differentiation

First, iPSCs and hESCs were induced to differentiate into NSCs as described above. The NSCs were then plated on 10-cm dishes coated with poly-*L*-ornithine (0.002%) (Sigma, P4957) and fibronectin (10 μg/mL; Millipore, 1918-FN-02 M, Boston, MA) in chemically defined and xeno-free astroglial medium. This medium contained DMEM/F12, 1 × N2 supplement, 1 × B27 supplement (without retinoic acid) (Thermo, 17504044), bone morphogenetic protein 4 (10 ng/ mL; Thermo, 120–05-5), and basic fibroblast growth factor (20 ng/ mL, Thermo, 100-18B-50UG) to promote directed astroglial differentiation and maturation over a 14-day period. Astrocytes were identified through immunostaining for S100 beta and glial fibrillary acidic protein (GFAP). The cells were passaged upon reaching 80%–90% confluence.

### Immunocytochemistry

DA neurons or astrocytes were cultured on 20-mm diameter glass coverslips in 12-well plates, and fixed with 4% paraformaldehyde for 10 min at room temperature. Following fixation, the cells were washed, permeabilized with 0.1% Triton X-100, and blocked with 10% BSA in PBS for 30 min prior to staining. The cells were incubated overnight at 4 °C with mouse anti-TH (1:1000; Abcam, ab129991, Cambridge, UK), rabbit anti-Tuj1 (1:1000; Abcam, ab18207), rabbit anti-GFAP (1:2000; Abcam, ab7260), and mouse anti-S100b (1:1000; Abcam, ab14849). Target antigens were then detected using species-appropriate fluorescence-conjugated secondary antibodies, such as Alexa Fluor 488 (Thermo, A-21202) or 555 (Thermo, A-31572). Visualization and imaging were performed with a Zeiss confocal microscope (Zeiss, LSM800, Oberkochen, Germany).

### Western blotting

DA neurons or astrocytes were seeded at a density of 0.6 × 10^5^/cm^2^ or 0.3 × 10^5^/cm^2^, respectively, in 35-mm culture dishes and harvested for analysis after they reached 90% confluence. Cells were lysed using a buffer composed of 5 mM EDTA, 150 mM sodium chloride, 25 mM Tris, and 1% Nonidet P-40 (pH 7.5) supplemented with a protease inhibitor cocktail (Sigma‒Aldrich, P8340). Protein concentrations in the supernatant were quantified using the bicinchoninic acid assay. Samples containing 30 μg of protein were loaded onto 10% sodium dodecyl sulfate‒polyacrylamide gel electrophoresis gels. Following electrophoresis, the proteins were transferred to polyvinylidene difluoride membranes (Millipore, IPVH00010), which were blocked in Tris-buffered saline with Tween 20 containing 5% (*w*/*v*) milk powder for 60 min at room temperature. The membranes were then incubated overnight at 4 °C with anti-GAPDH (1:2000, Sigma‒Aldrich, G8795), anti-TH (1:1000, Abcam, ab129991), anti-Tuj1 (1:1000, Abcam, ab18207), anti-GFAP (1:1000, Abcam, ab7260), anti-S100b (1:1000, Abcam, ab14849), anti-DRP1 (1:2000, Proteintech, 12957–1-AP, Wuhan, Chin), anti-p616 DRP1 (1:1000, CST, #3455, Boston, MA), anti-STX17 (1:2000, Proteintech, 17815–1-AP), anti-SNAP23 (1:1000, Abcam, ab131242), or anti-VAMP3 (1:1000, Abcam, ab283313). The membranes were subsequently incubated with horseradish peroxidase-conjugated secondary antibodies, and visualization was enhanced using an enhanced chemiluminescencedetection system (GE Healthcare, NA931 for anti-mouse, NA934 for anti-rabbit, or NA935 for anti-rat, Chicago, IL). Optical densities were quantified with the ImageJ software (NIH, Bethesda, MD).

### Establishment of the coculture system

In one set of experiments, iPSC-derived astrocytes (6 × 10^4^/cm^2^) were carefully overlaid onto iPSC-derived DA neurons (6 × 10^4^/cm^2^, cultured in the DA neuron maturation medium) to establish a coculture system that maintained a 1:1 neuron-to-astrocyte ratio. To assess cell survival and neurite length, three random fields (20 × objective) were imaged, and the neurons were counted and averaged. Three to five independent experiments were conducted.

### Rotenone-induced neurotoxicity

Immature neurons were plated in 12-well plates and cultured in the DA neuron maturation medium for 7–11 days as described. Upon maturation, the astrocytes were overlaid at a 1:1 ratio for 24 h of coculture, followed by treatment with varying concentrations (0, 50, 100, 200 nM) of rotenone for 24 h.

### Assessment of neuronal damage

Cell survival was evaluated by counting TH-positive neurons after different treatments using the ImageJ software (NIH, Bethesda, MD). Each merged image was opened in ImageJ, and manual counting was performed using the multipoint macro add-in. Neurite lengths were measured using ImageJ, where Tuj1 and TH double-stained images were analyzed with the segmented line tool. Each curved neurite was measured as a series of short, straight segments, and the total length was determined as the sum of all the segments. Three random fields (20 × objective) were imaged and analyzed.

### Mitochondria tracking

iPSC-derived astrocytes were incubated with MitoTracker probes (Invitrogen/Molecular Probes; 100 nM, M7514, Waltham, MA) for 30 min, allowing passive diffusion of the dye through the cell membrane and subsequent accumulation in active mitochondria, according to the manufacturer's instructions [17, 30]. Once labeled with MitoTracker, the movement and translocation of astrocyte mitochondria into other cells were observed using a confocal fluorescence microscope (Zeiss, LSM800).

### Flow cytometry

Standard flow cytometry analysis was performed using a BD LSR II (BD Biosciences, Franklin Lake, NJ), following previously described protocols. Astrocyte-conditioned medium (ACM) was collected from the MitoTracker-labeled astrocytes and filtered through a 1.2-μm syringe filter. Particles containing mitochondria were subsequently sorted via FACS analysis using size-adjusted gating optimization.

### Transient transfection with siRNA

Control siRNA and target siRNA (GenePharma, Shanghai, China) were used for transient transfection. The siRNA sequences were as follows: human *DRP1* siRNA (siDRP1#1: 5′-AAGCAGAAGAATGGGGTAAAT-3′ and siDRP1#2: 5′-GGAGCCAGCTAGATATTAA-3′), human *VAMP3* siRNA 5′-TCAAGCTTACCTACTGTTA-3′, human *SNAP23* siRNA 5’-GAGUCUGGCAAGGCUUAUATT-3′, and human *STX17* siRNA 5′-GATAGTAATCCCAACAGACC-3′. A human nontargeting siRNA (5′-UGGUUUACAUGUCGACUAA-3′) was used as a control. Neurons were transiently transfected with siRNA using Lipofectamine 3000 transfection reagent (Invitrogen) following the manufacturer's instructions. The efficiency of target gene knockdown at the protein level was assessed by Western blotting 24 h post-transfection.

### Statistical analysis

All data are presented as the mean ± SEM. Unpaired *t* test was used for comparisons between two experimental groups. One-way ANOVA followed by Tukey's test was used for multiple comparisons. Statistical analyses were conducted using the Prism 5 software (GraphPad). *P* < 0.05 was considered as statistically significant.

## Results

### Differentiation of iPSCs into astrocytes and DA neurons

**Fig. 1:**
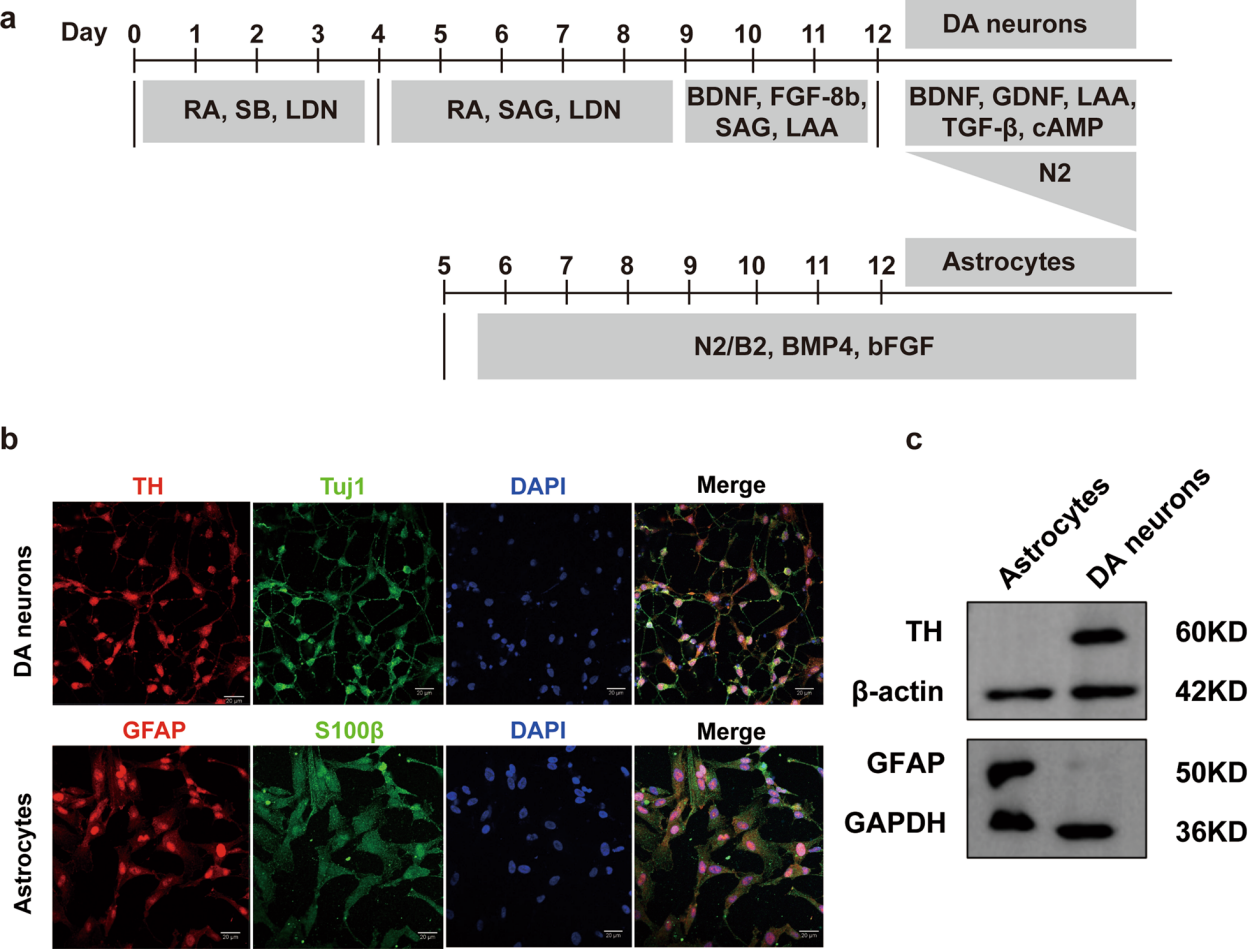
Generation of human iPSC-derived DA neurons and astrocytes. **a** Flow chart for the differentiation of DA neurons and astrocytes from human iPSCs. **b** Representative confocal immunocytochemistry of the DA neuron-specific markers TH (red) and Tuj1 (green), as well as the astrocyte-specific markers GFAP (red) and S100β (green). **c** Western blotting for TH in DA neurons and GFAP in astrocytes. Three independent experiments were performed (*n* = 3). Scale bars, 20 μm

iPSCs were generated from PBMCs of a healthy donor and a PD patient with a *LRRK2* G2019S mutation, who is the first documented PD case harboring the *LRRK2* G2019S mutation in China by our team [31]. According to the genetic testing report, the PD patient is negative for other known monogenic causes of PD, and is heterozygous for the *LRRK2* G2019S mutation. Karyotype analysis of iPSCs revealed a normal 46XY karyotype without chromosomal abnormalities (Fig. S2a, b). Initially, the dual inhibitors SB431542 and Noggin were utilized to inhibit the SMAD signaling pathway, inducing the neuralization of iPSCs into NSCs. By supplementing the cultures with various small molecules, differentiation into astrocytes and DA neurons was achieved. Immunofluorescence revealed that astrocytes expressed GFAP and S100 calcium-binding protein β (S100β), while DA neurons expressed TH and Tuj1 (Fig. 1b). Western blotting also confirmed the successful differentiation of iPSCs into astrocytes and DA neurons (Fig. 1c).

### The *LRRK2* G2019S mutation impairs the transfer of mitochondria from astrocytes to DA neurons under rotenone exposure

**Fig. 2:**
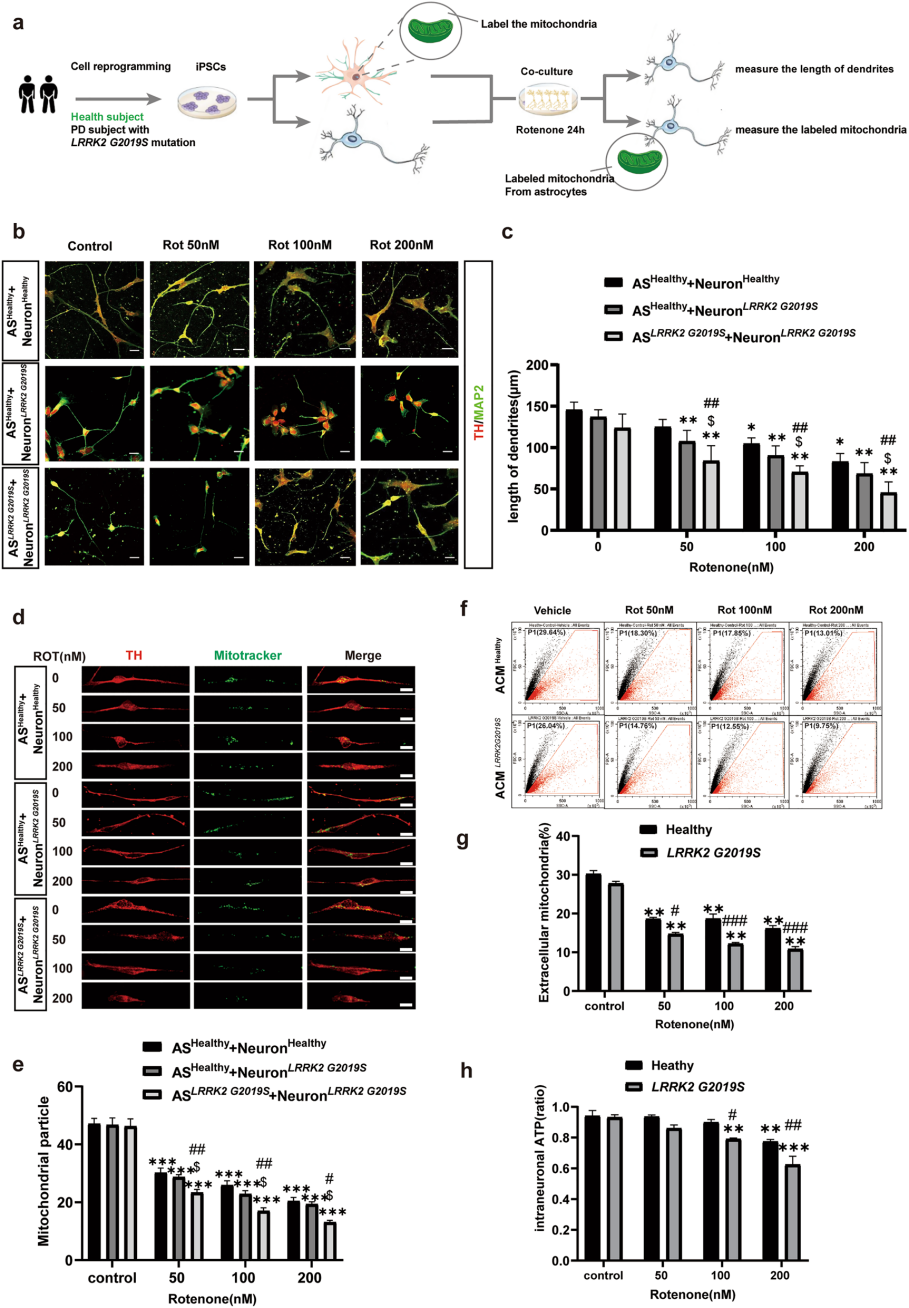
The *LRRK2* G2019S mutation combined with rotenone contributes to DA neuron degeneration and impairs the transfer of mitochondria from astrocytes to DA neurons. **a** Schematic of the astrocyte–neuron coculture experiments. **b** Representative TH and MAP2 immunostaining to show DA neurons in different coculture systems treated with different concentrations of rotenone. **c** Quantification of dendritic lengths of the DA neurons (TH and MAP2 double staining). **d** Mitochondria in astrocytes were labeled with MitoTracker (green) before they were cocultured with DA neurons. After 24 h of coculture, immunostaining revealed that astrocytic mitochondria were present in the DA neurons. Upon exposure to different concentrations of rotenone, the quantity of labeled mitochondria in DA neurons differed. **e** Quantification of astrocytic mitochondria in DA neurons in the different groups. **f** ACM was collected, and the number of mitochondria labeled with MitoTracker was determined via FACS analysis using size-adjusted gating optimization. **g** Quantification of the astrocytic mitochondria in ACM as measured by flow cytometry. **h** Measurement of intraneuronal ATP levels in DA neurons cultured with ACM collected from rotenone-treated astrocytes. Three independent experiments were performed (*n* = 3). **c**, **e** ^*^P < 0.01 vs. control, ^**^P < 0.005 vs. control, ^***^P < 0.001 vs. control, ^#^*P* < 0.01 vs. AS^Healthy^ + Neuron^Healthy^, ^##^*P* < 0.005 vs. AS^Healthy^ + Neuron^Healthy^, ^$^*P* < 0.01 vs. AS^Healthy^ + Neuron^LRRK2 G2019S^. **g**, **h** **P < 0.005 vs. control, ***P < 0.001 vs. control, ^#^*P* < 0.01 vs. Health, ^##^*P* < 0.005 vs. Health, ^###^*P* < 0.001 vs. Health. Mean ± SEM, two-way ANOVA followed by Tukey's HSD test. Scale bars, 20 μm

Our previous report indicated that astrocytes could protect neurons under rotenone exposure by donating mitochondria to DA neurons, suggesting a novel endogenous protective mechanism based on mitochondrial transfer and supplementation [32]. Here, we investigated whether this protective phenomenon still exists in individuals with the *LRRK2* G2019S mutation. The “astrocyte-DA neuron” cocultures from both the healthy individual and the patient with the *LRRK2* G2019S mutation were established to mimic the adjacent growth relationship between neurons and astrocytes in the brain (Fig. 2a). Rotenone (50, 100, and 200 nM) was used to model the effects of environmental toxins. Microtubule-associated protein 2 (MAP2) is highly expressed in neurons, and TH is highly expressed in DA neurons; thus, we used MAP2 co-staining with TH to visualize the morphology of DA neurons [16]. As shown in Fig. 2b, compared with the control group, rotenone induced significant synaptic shortening, which was most pronounced at a rotenone concentration of 200 nM. Particularly, compared with the control group, the 200 nM rotenone treatment led to a nearly 60% reduction of dendrite length in the mutant coculture group (Astrocyte^*LRRK2* G2019S^ + Neuron^*LRRK2* G2019S^). In contrast, the dendritic length of DA neurons in the wild-type coculture system (Astrocyte^healthy^ + Neuron^healthy^) was 44% lower at a concentration of 200 nM rotenone compared with the control group (Fig. 2b, c). This difference indicates a reduced capacity to withstand environmental toxins in the mutant coculture system. To investigate whether astrocytes influence the activity of DA neurons, we cocultured wild-type astrocytes with *LRRK2* G2019S-mutant DA neurons (Astrocyte^healthy^ + Neuron^*LRRK2* G2019S^). We found that the damage to DA neurons was less severe than it was in the mutant coculture system (Astrocyte^*LRRK2* G2019S^ + Neuron^*LRRK2* G2019S^). For instance, 200 nM rotenone shortened the length of DA neuron dendrites by 50%, indicating milder damage than that observed in the mutant group (63.75% reduction) (Fig. 2b, c).

Further investigations were conducted to determine whether mitochondrial transfer between astrocytes and DA neurons was impaired in the coculture system exposed to rotenone. MitoTracker staining revealed no baseline differences in mitochondrial levels between wild-type and mutant astrocytes (Fig. 2d, e). Upon exposure to rotenone, the quantity of labeled astrocytic mitochondria in DA neurons decreased in a dose-dependent manner. In the mutant coculture system, the mitochondrial count decreased to 70% in the presence of 200 nM rotenone, which was more pronounced than the decrease of 60% in the wild-type coculture system (Fig. 2d, e). Compared with the mutant coculture system, mitochondrial transfer in the Astrocyte^healthy^ + Neuron^*LRRK2* G2019S^ system showed a milder reduction of mitochondrial transfer. In particular, after treatment with 200 nM rotenone, the number of mitochondrial transfer particles decreased to 56%, showing a significantly lower reduction compared to the mutant coculture system (70% reduction) (Fig. 2e). These findings suggest that *LRRK2* G2019S may mitigate the mitochondrial transfer between astrocytes and DA neurons.

Flow cytometry experiments yielded similar results. ACMs were collected, and the number of mitochondria labeled with MitoTracker was quantified. The results demonstrated a decrease in mitochondrial numbers in the ACM with increasing rotenone concentration, which is consistent with our previously reported results [32]. Furthermore, 200 nM rotenone induced a more significant decrease of mitochondria number in ACM^LRRK2 G2019S^ (62.6% reduction) compared to ACM^healthy^ (56.1% reduction). (Fig. 2f, g). This intriguing phenomenon suggests that both rotenone exposure and the *LRRK2* G2019S mutation impair mitochondrial transfer during the stage of mitochondrial release from astrocytes. Moreover, when DA neurons from both healthy and *LRRK2* G2019S participants were cultured with corresponding ACM^healthy^ and ACM^LRRK2 G2019S^, they exhibited reduced intracellular ATP levels following exposure to rotenone. Notably, DA neurons harboring the *LRRK2* G2019S mutation exhibited significantly greater impairment in ATP production (Fig. 2h). These results suggest that *LRRK2* G2019S enhances the sensitivity of neurons to environmental toxin damage through a reduction in mitochondrial transfer from astrocytes.

### The membrane fusion-related protein STX17 is involved in mitochondrial output in a SNARE-independent mechanism

**Fig. 3:**
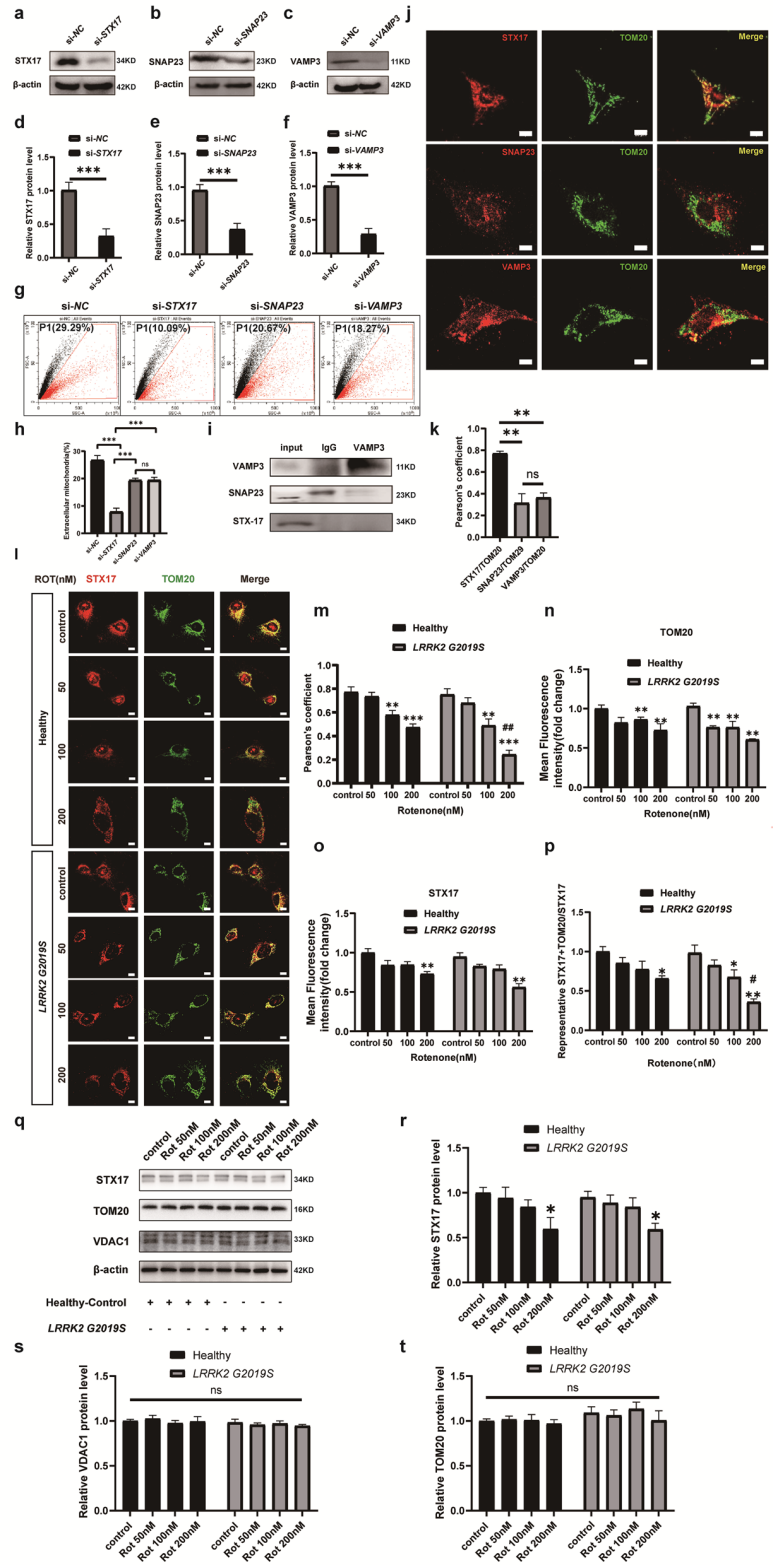
STX17 is involved in astrocytic mitochondrial output through a SNARE-independent mechanism. **a**–**f** Western blots showing the efficient knockdown of STX17, SNAP23 and VAMP3 expression and quantification. **g, h** Quantification of astrocyte-derived mitochondria in ACM via flow cytometry. **i** Immunoprecipitation results showing the interactions among STX17, SNAP23 and VAMP3. **j** Immunofluorescence analysis of the colocalization of TOM20 with STX17, VAMP3 and SNAP23. **k** Colocalization of STX17, SNAP23 and VAMP3 with TOM20 analyzed through Pearson’s correlation coefficients. **l** Immunofluorescence analysis revealed colocalization of TOM20 with STX17 in healthy and *LRRK2* G2019S astrocytes treated with different doses of rotenone. **m** Colocalization of STX17 with TOM20 according to Pearson’s correlation coefficients in healthy and mutant astrocytes treated with various doses of rotenone. **n**, **o** Quantification of the immunofluorescence intensities of STX17 and TOM20. **p** Quantification of STX17 and TOM20 colocalization relative to STX17 signal. **q**–**t** Western blots for STX17, VDAC1 and TOM20 in healthy and mutated astrocytes treated with different doses of rotenone, and quantification. These experiments were performed in pure astrocyte culture. Three independent experiments were performed (*n* = 3). **d**–**f** ^***^P < 0.001 vs. si-*NC*, *t*-test. **h** ^***^P < 0.001, one-way ANOVA. **k** ^**^P < 0.005, one-way ANOVA. **n, m, o, p, r, s, t** ^*^P < 0.01, ^**^P < 0.005, ^***^P < 0.001 vs. control, ^#^*P* < 0.01, ^##^*P* < 0.005 vs. Health; two-way ANOVA analysis followed by Tukey’s HSD test. The results are presented as the mean ± SEM. Scale bars, 10 μm

STX17 is a key protein involved in membrane fusion. Recent studies have indicated its role in regulating mitophagy by mediating mitochondrial membrane fusion [21, 34]. Therefore, we hypothesized that STX17 may be involved in the membrane fusion-related mitochondrial output. STX17 is a member of the SNARE protein family, and SNARE-dependent fusion involves a classic exocytosis regulatory complex mechanism that plays a central role in the exocytosis of substances or organelles such as neurotransmitters, lysosomes, and exosomes [35]. VAMP3 and SNAP23, two other SNARE family proteins abundantly expressed in astrocytes, are recognized as key regulators of astrocyte exocytosis [36]. We knocked down expression of STX17, VAMP3, and SNAP23 in astrocytes using RNA interference technology, and the quantity of astrocyte-derived mitochondria in ACM was assessed via flow cytometry. Western blot verified that the protein levels were significantly reduced after RNA interference treatment (Fig. 3a–f), and the flow cytometry analysis revealed a reduction in mitochondrial count in ACM after knockdown of these proteins, with *STX17* knockdown inducing the most pronounced decrease (70.70% reduction). In contrast, *VAMP3* and *SNAP23* knockdown resulted in moderate decreases (27.4% and 27.3%, respectively) (Fig. 3g, h). Immunoprecipitation indicated that VAMP3 does not bind to STX17 or SNAP23 (Fig. 3i). Immunofluorescence analysis further demonstrated that STX17 colocalizes with the mitochondrial outer membrane protein TOM20, whereas VAMP3 and SNAP23 did not colocalize with TOM20 (Fig. 3j). These results suggest that STX17 is closely associated with the mitochondrial outer membrane and may be related to mitochondrial transfer independent of the SNARE complex formation with VAMP3 and SNAP23.

We then assessed the spatial relationship between STX17 and the mitochondrial outer membrane in wild-type and *LRRK2* G2019S-mutant astrocytes treated with varying concentrations of rotenone. The results revealed a decrease in the colocalization of STX17 and TOM20 with increasing rotenone concentrations, and the *LRRK2* G2019S-mutant astrocytes displayed a more pronounced reduction compared to the wild-type astrocytes (Fig. 3l). To further assess mitochondrial quantity and mitophagy levels and thereby evaluate whether the degree of colocalization objectively reflects the ability of STX17 to target mitochondria, we first quantified the fluorescence intensities of STX17 and TOM20, followed by Western blot analysis of TOM20, VDAC1, and STX17. TOM20 fluorescence and protein level did not significantly differ between genetic backgrounds or across varying rotenone concentrations (Fig. 3n, q, t). We also revealed no significant changes in VDAC1 protein levels (Fig. 3q, s). As TOM20 and VDAC1 are outer mitochondrial membrane proteins that partially reflect mitochondrial quantity, these results indicate that the mitochondrial mass was stable even after 200 nM rotenone treatment in the mutants. However, rotenone (200 nM) significantly reduced STX17 fluorescence intensity in both the wild-type (27% reduction) and *LRRK2* G2019S-mutant astrocytes (41% reduction) (Fig. 3l, o), with no differences between the genotypes. Western blot analysis verified this result (Fig. 3q, r).

Given that a reduction in STX17 protein level may also affect its degree of colocalization with mitochondria, we further analyzed the colocalization. The results revealed decreased STX17-TOM20 colocalization under rotenone (50, 100, and 200 nM) treatment (Fig. 3p). These findings indicate that the ability of STX17 to localize at the mitochondrial outer membrane was reduced at rotenone concentrations of 50 and 100 nM. In response to treatment with 200 nM rotenone, the decreased level of STX17 protein combined with impaired targeting ability collectively caused a significant reduction in its colocalization.

Since rotenone has been reported to activate mitophagy, which may affect mitochondrial degradation in donor astrocytes and thereby confound our analysis, we also measured mitophagy-related proteins to examine mitophagy activation. Western blotting revealed that under rotenone treatment, there were no significant differences in PINK1, Parkin, or their phosphorylation levels between wild-type and *LRRK2* G2019S astrocytes (Fig. S5a–c).

Collectively, these findings show that the mitochondrial quantity remained constant, with no increase in mitochondrial degradation, indicating a relatively healthy mitochondrial state in both Astrocyte^healthy^ and Astrocyte^*LRRK2* G2019S^ with or without rotenone exposure. Furthermore, both *LRRK2* G2019S mutation and rotenone exposure reduced the colocalization of STX17 with mitochondria, indicating a close relationship between STX17 localization and mitochondrial transfer.

### Mitochondrial Drp1 plays a role in mitochondrial transfer

**Fig. 4:**
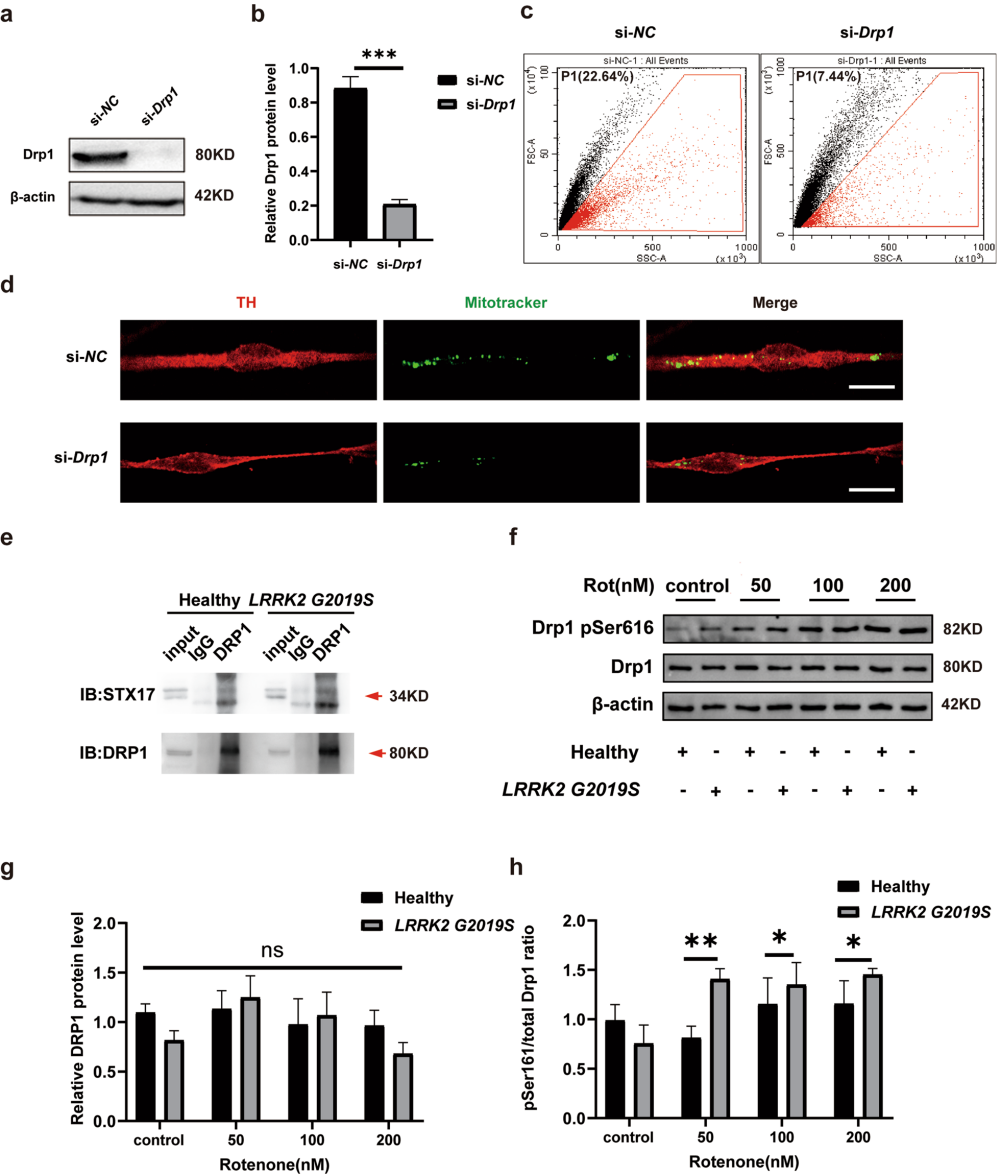
Drp1 participates in STX17-dependent mitochondrial transfer. **a**, **b** Western blots showing knockdown of Drp1, and quantification. **c** Flow cytometry was used to measure the number of mitochondrial particles in ACM with and without Drp1. **d** Immunofluorescence for astrocytic mitochondria in DA neurons in a coculture system with and without Drp1. **e** Immunoprecipitation results showing the interactions between STX17 and Drp1 in healthy and mutant astrocytes. **f**–**h** Western blots showing the amount of total Drp1 and Drp1 pSer616 in pure cultures of healthy and mutant astrocytes treated with different doses of rotenone, and quantification. Three independent experiments were performed (*n* = 3). **b** ***P < 0.001, *t*-test. **g**, **h** *P < 0.01, **P < 0.005, two-way ANOVA followed by Tukey's HSD test. Mean ± SEM. Scale bars, 20 μm

Drp1 is a highly conserved cytosolic GTPase that belongs to the dynamin superfamily and is recognized as the classical key protein that regulates mitochondrial fission [37]. Drp1 activity is regulated through posttranslational modifications such as phosphorylation and dephosphorylation, thereby influencing mitochondrial dynamics. Recent studies have implicated an interaction between Drp1 and STX17 in the dynamic processes of mitochondrial fusion [21, 38]. Our previous findings indicated that STX17 is involved in mitochondrial export. Therefore, we next investigated whether Drp1 regulates the colocalization of STX17 with mitochondria and affects mitochondrial transfer through the phosphorylation of Drp1. We knocked down *DRP1* in astrocytes, which was confirmed by Western blotting (Fig. 4a, b). Flow cytometry analysis revealed a 67% reduction of mitochondrial particle number released in the ACM after *DRP1* knockdown (Fig. 4c). Moreover, *DRP1* knockdown in the coculture system (Astrocyte^healthy^ + Neuron^healthy^) significantly decreased the mitochondrial transfer efficiency (Fig. 4d). Immunoprecipitation also confirmed that STX17 and Drp1 are colocalized in both wild-type and *LRRK2* G2019S mutation-expressing astrocytes (Fig. 4e). These results support our hypothesis that Drp1 may be involved in mitochondrial transfer and that its activity may occur through interactions with STX17.

### *LRRK2* G2019S mutation combined with rotenone treatment affects the phosphorylation of Drp1 at Ser616

**Fig. 5:**
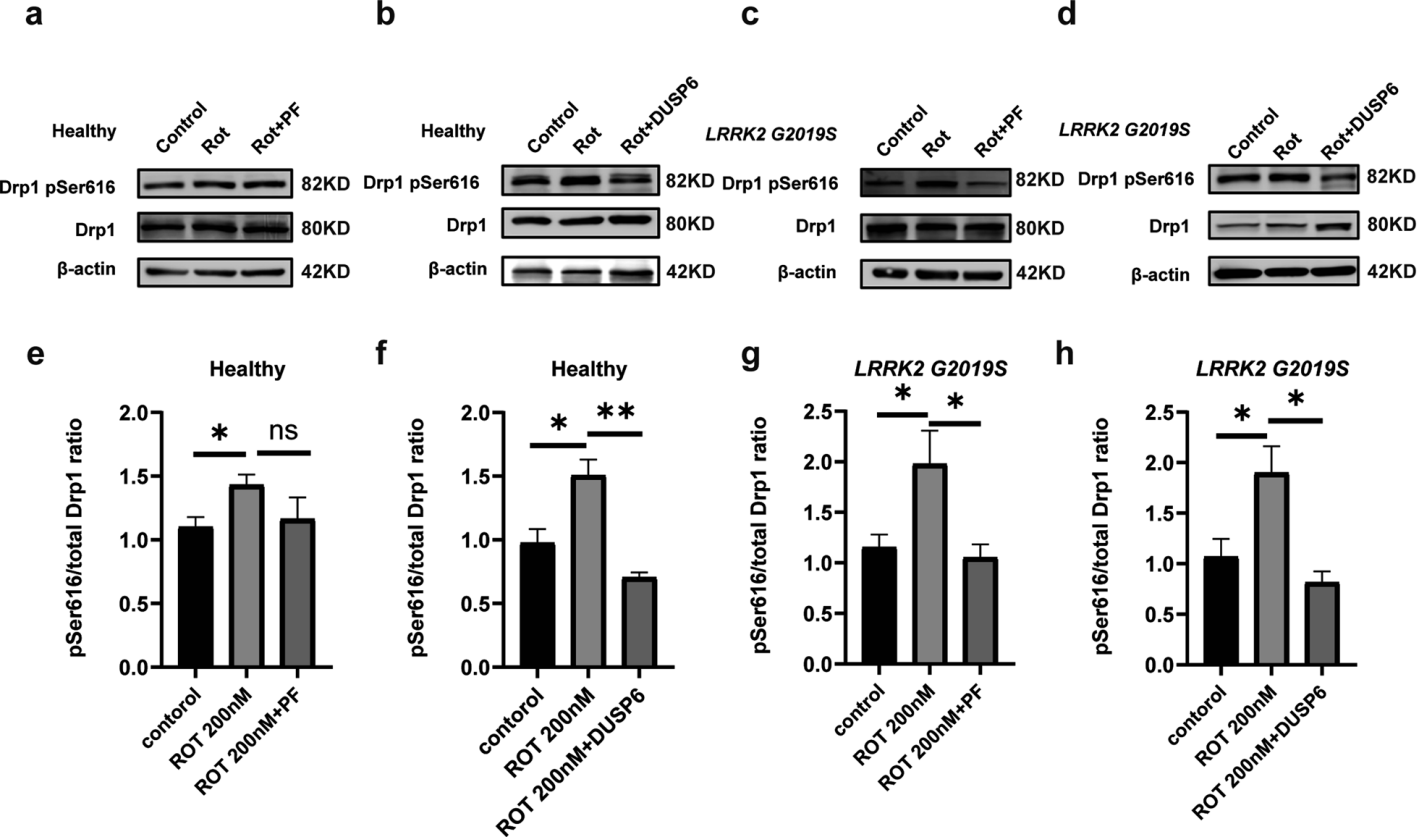
The Drp1 inhibitor DUSP6 reduces the level of Drp1 pSer616 in both wild-type and *LRRK2* G2019S astrocytes. **a**, **c** Western blots for total Drp1 and Drp1 pSer616 in control, 200 nM rotenone, and 200 nM rotenone + PF-06447475 groups. **b, d** Western blots for total Drp1 and Drp1 pSer616 in the control, 200 nM rotenone, and 200 nM rotenone + DUSP6 groups. **e**–**h** Quantification of the protein levels of Drp1 and Drp1 pSer616. These experiments above were conducted in pure astrocyte cultures. Three independent experiments were performed (*n* = 3). **e–h** ^*^P < 0.01, ^**^P < 0.005, one-way ANOVA followed by Tukey’s HSD test. Mean ± SEM

Drp1 activity is particularly dependent on its phosphorylation status, with Ser616 phosphorylation linked to enhanced mitochondrial fission [39]. To further elucidate the role of Drp1 in mitochondrial transfer, both total and phosphorylated Drp1 levels in astrocytes were analyzed by Western blotting (Fig. 4f). The Ser616-phosphorylated form of Drp1 increased in a rotenone concentration-dependent manner, with a more pronounced upregulation observed in astrocytes carrying the *LRRK2* G2019S mutation compared to wild-type astrocytes. The mRNA and protein levels of total LRRK2 remained unchanged in healthy and mutant astrocytes, but its phosphorylation at Ser1292 was increased (Fig. S4a–c). These findings confirm that LRRK2 kinase activity, rather than its amount, plays a role in Drp1 phosphorylation. Specifically, compared with baseline, 200 nM rotenone increased the levels of phosphorylated Drp1 Ser616 by 1.9-fold and 2.5-fold in wild-type and *LRRK2* G2019S astrocytes, respectively, while the total Drp1 levels remained stable across the different conditions (Fig. 4f–h). These results suggest that both the *LRRK2* G2019S mutation and rotenone treatment contribute to Drp1 activity. Next, we treated astrocytes with the LRRK2 kinase inhibitor PF-06447475 (0.5 μM, 24 h) and the Drp1 phosphorylation inhibitor DUSP6 (0.25 μg/μl, 24 h) in the presence of rotenone, and assessed Drp1 phosphorylation levels. PF-06447475 effectively reduced Drp1 phosphorylation (27% reduction) in *LRRK2* G2019S-mutant astrocytes by inhibiting *LRRK2* activity (Fig. 5c, g), but had no significant effect on normal astrocytes (Fig. 5a, e). Conversely, DUSP6 reduced Drp1 phosphorylation in both wild-type (53% reduction) and *LRRK2* G2019S-mutant (57% reduction) astrocytes (Fig. 5b, d, f, h). The dephosphorylation function of DUSP6 (0.25 μg/μL, 24 h) on Drp1 Ser616 was also validated (Fig. S3a, b).

### The phosphorylation of Drp1 influences the displacement of STX17 from mitochondria

**Fig. 6:**
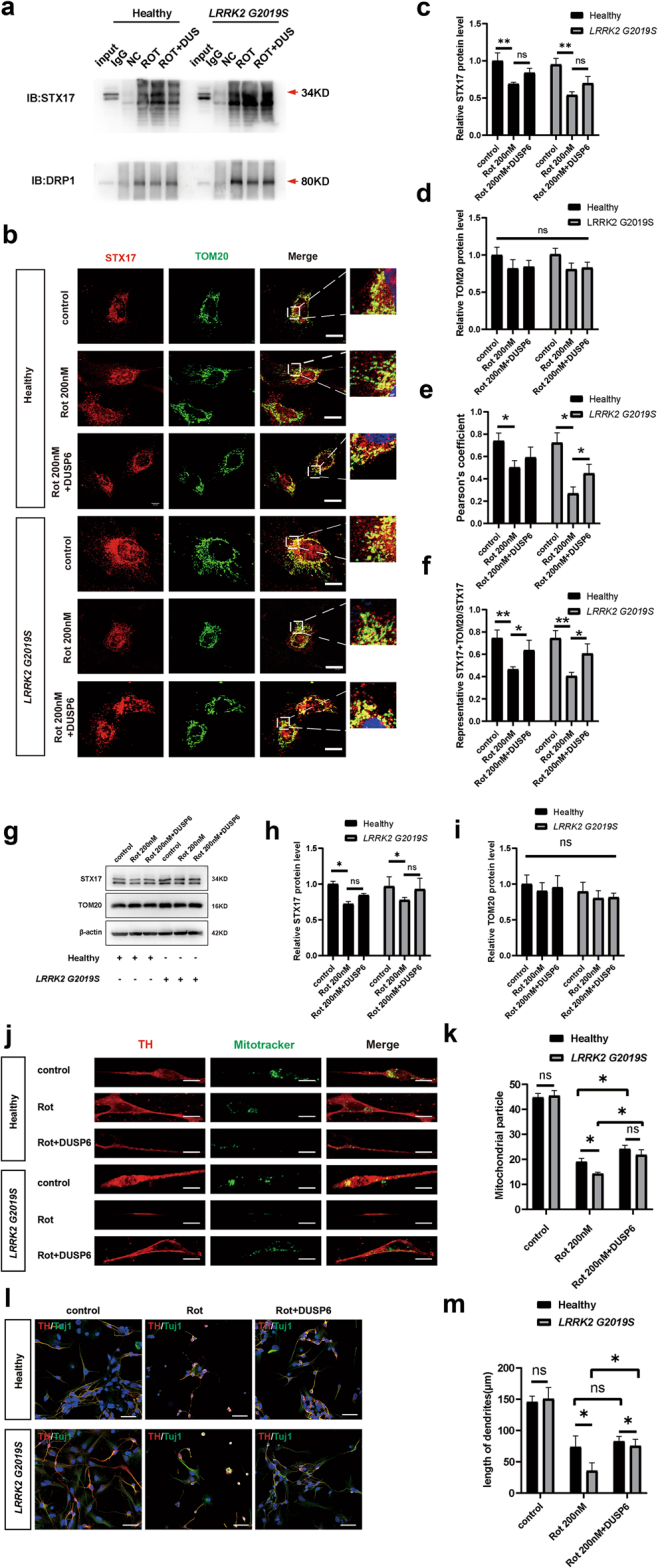
DUSP6 rescues impaired mitochondrial transfer and DA neuron damage in both wild-type and *LRRK2* G2019S-expressing astrocytes. All the experiments below included the following groups: control, 200 nM rotenone, and 200 nM rotenone + DUSP6, in both healthy and mutant models. **a** Immunoprecipitation for analyzing interactions between STX17 and Drp1 in pure astrocyte cultures. **b** Immunofluorescence analysis for the colocalization of TOM20 with STX17 in pure astrocyte cultures. **c, d** Quantification of the immunofluorescence intensities of STX17 and TOM20 in pure astrocyte cultures. **e** The colocalization of STX17 with TOM20 in pure astrocyte cultures was quantified through Pearson’s correlation coefficient. **f** Quantification of STX17 and TOM20 colocalization relative to STX17 signal in pure astrocyte cultures. **g**–**i** Western blots for STX17 and TOM20 in pure astrocyte cultures, and quantification. **j** Immunofluorescence staining for astrocytic mitochondria in the DA neurons in the co-culture system. **k** Quantification of mitochondrial immunofluorescence in DA neurons. **l** Representative confocal images of DA neurons in the co-culture system. **m** Quantification of the dendritic lengths of the DA neurons in the groups described above. Three independent experiments were performed (*n* = 3). **c–f, h, i, k, m** ^*^P < 0.01, ^**^P < 0.005, two-way ANOVA followed by Tukey’s HSD test. Mean ± SEM. Scale bars, 20 μm for **b**, **j**, 75 μm for **l**

Coimmunoprecipitation confirmed that treatment with 200 nM rotenone significantly reduced the interaction between Drp1 and STX17, whereas treatment with DUSP6 (0.25 μg/mL, 24 h) effectively enhanced their interaction (Fig. 6a). Further validation was conducted to determine whether the phosphorylation of Drp1 Ser616 regulates mitochondrial transfer by altering the positioning of STX17 under rotenone treatment in wild-type and mutant astrocytes. Immunofluorescence analysis showed that under the 200 nM rotenone treatment, STX17 detached from mitochondria in both wild-type and *LRRK2* G2019S-mutant astrocytes, with this effect being particularly pronounced in *LRRK2* G2019S-mutant astrocytes. Notably, DUSP6 not only inhibited Drp1 Ser616 phosphorylation, but also prevented the detachment of STX17 from mitochondria (Fig. 6b). Notably, quantitative analysis revealed that treatment with 200 nM rotenone significantly reduced the colocalization of STX17 with mitochondria (37.7% decrease in the wild-type astrocytes and 46% decrease in the mutant astrocytes), whereas treatment with DUSP6 (0.25 μg/mL) markedly increased the degree of STX17–mitochondria colocalization (37% increase in the wild-type astrocytes and 50% increase in the mutant astrocytes) (Fig. 6c–f). Western blotting for TOM20 and STX17 (Fig. 6g–i) supported the fluorescence results. These findings further demonstrated that rotenone (200 nM) reduced and that DUSP6 (0.25 μg/mL) increased the mitochondrial targeting ability of STX17. In addition, although the STX17 protein level decreased with 200 nM rotenone treatment, STX17 still had ability to localize to mitochondria. These data suggest that the phosphorylation of Drp1 Ser616 leads to the detachment of STX17 from mitochondria, resulting in the loss of the role of STX17 in guiding mitochondrial transfer. This effect is reversed by the Drp1 phosphorylation inhibitor DUSP6.

### DUSP6 can rescue impaired mitochondrial transfer and DA neuron damage

In both the mutant and wild-type co-culture systems, 200 nM rotenone reduced mitochondrial transfer efficiency (46% reduction in the wild-type group and 52% reduction in the mutant group) (Fig. 6j, k) and shortened dendrites (43% and 50%, respectively) (Fig. 6l, m). Notably, DUSP6 treatment improved mitochondrial transfer efficiency (27% increase in the wild-type group and 53% increase in the mutant group) (Fig. 6j, k) and mitigated neuronal damage (12% and 111% increase of dendritic length, respectively) (Fig. 6l, m).

## Discussions

Mitochondrial transfer has emerged as a crucial mechanism of intercellular communication and signal transduction. Previous studies have reported that the transport of mitochondria from astrocytes to neurons may serve as an endogenous neural repair mechanism in PD [40, 41]. In this study, we report for the first time the mitochondrial transfer dysfunction in the astrocyte-DA neuron coculture system derived from a PD patient with *LRRK2* G2019S mutation. Moreover, this study provides novel insights into the synergistic pathogenic mechanisms of genetic and environmental factors and identifies mitochondrial transfer as a new therapeutic target for PD intervention.

*LRRK2* mutations are the most common genetic risk factors for PD. Among these, the G2019S variant is the most prevalent globally, reaching an incidence of approximately 40% in North African Berber populations with PD [42–44]. However, it remains rare in the Han Chinese population. In 2018, our team reported the first documented PD case harboring the *LRRK2* G2019S mutation in China [31]. A healthy volunteer and one PD patient donated cells for iPSC experiments, offering a valuable opportunity to study the distinct pathophysiological effects of this mutation in Chinese genetic background.

A coculture system comprising human iPSC-derived DA neurons and astrocytes was established to simulate the neuronal–glial cell symbiotic environment in the brain (Fig. 2a). The data demonstrated that neurons carrying the *LRRK2* G2019S mutation exhibit increased sensitivity to environmental toxins (Fig. 2b, c). Notably, our study revealed that both rotenone exposure and *LRRK2* G2019S mutation impaired mitochondrial transfer from astrocytes to DA neurons, highlighting that reduced mitochondrial transfer contributes to DA neuronal damage (Fig. 2d, e). We propose that mitochondrial transfer dysfunction is a key pathogenic mechanism of the *LRRK2* G2019S mutation.

We next investigated the molecular mechanisms underlying mitochondrial transfer and how the *LRRK2* G2019S mutation and the environmental toxin rotenone affect this process. In our previous work, we did not find formation of tunneling nanotubes [32], the most commonly established structure that facilitates mitochondrial transfer [17, 45], between DA neurons and astrocytes during mitochondrial transfer. However, we did detect astrocyte-derived mitochondria in ACM, which is consistent with previous reports [16, 32]. These findings strongly suggest that astrocytes can release mitochondria extracellularly, which are subsequently taken up by neurons, resembling “endocytic” and “exocytic” processes. Therefore, in this study we focused on astrocytic “exocytosis” of mitochondria, which is the critical initial step of mitochondrial transfer.

Given that the SNARE mechanism mediates most membrane fusion events, including exocytosis, the potential roles of key SNARE proteins in driving mitochondrial exocytosis were investigated [21, 46]. In the classic SNARE mechanism, three types of SNARE family proteins interact to form stable tetramers, providing the structural basis for membrane fusion [47]. Our data demonstrated that STX17 is closely associated with mitochondrial release from astrocytes, whereas the other two proteins in the complex, SNAP23 and VAMP3 (which are highly expressed in astrocytes and are key regulators of astrocytic exocytosis), do not colocalize with mitochondria or participate in mitochondrial release [48]. These findings suggest that STX17 may be involved in mitochondrial exocytosis through a non-SNARE-dependent mechanism (Fig. 3a–k).

STX17 is involved in multiple non-SNARE-dependent membrane fusion mechanisms. STX17 plays a role in autophagic lysosome formation [49], and also mediates “mitochondria‒lysosome” fusion by anchoring its transmembrane domain to the outer mitochondrial membrane, thereby regulating mitophagy and mitochondrial degradation [21]. Notably, rotenone activates mitophagy [50]. Thus, we investigated the effects of rotenone and *LRRK2* G2019S mutation on mitophagy and mitochondrial abundance, and whether STX17 promotes mitochondrial degradation. We found that at rotenone concentrations of 50–200 nM, neither wild-type nor *LRRK2* G2019S hiPSC-derived astrocytes exhibited mitophagy, mitochondrial degradation, or significant changes in mitochondrial levels (Figs. 3q–t, 6a–c). Nevertheless, since mitophagy and mitochondrial transfer involve mitochondrial dynamics, whether there is an overlap between their regulatory mechanisms remains unclear. Although mitochondrial transfer may activate mitophagy in recipient cells [33], the donor cell mitophagy is poorly understood. Future studies using wider ranges of rotenone concentrations are needed to investigate the relationship between these two processes and advancing understanding of mitochondrial transfer.

Next, we investigated how STX17 is regulated in the model and whether other proteins are involved. The *LRRK2* G2019S mutation enhances the kinase activity of LRRK2, increasing protein phosphorylation [51]. Similarly, rotenone phosphorylates downstream proteins, contributing to its pathogenesis. While the function of STX17 is linked to its localization, no studies have reported phosphorylation-dependent regulation of its activity. The fission-related protein Drp1, located on the outer mitochondrial membrane, mediates mitochondrial dynamics and is regulated by phosphorylation [52]. Arasaki et al. reported that hunger signaling abrogates the interaction between STX17 and Drp1, causing STX17 to detach from mitochondria [21], suggesting that Drp1 may influence the localization and functional performance of STX17. Drp1 has multiple phosphorylation sites. The *LRRK2* G2019S mutation specifically increases the phosphorylation of Drp1 at Ser616 [53]. The phosphorylation at Ser616 can regulate Drp1 intracellular localization [54, 55]. In this study, we found that both rotenone and *LRRK2* G2019S mutation increased Drp1 Ser616 phosphorylation, causing STX17 detachment from mitochondria.

To further investigate whether Drp1 phosphorylation affects mitochondrial transfer, we used the *LRRK2* inhibitor PF-06447475 and the Drp1 phosphorylation inhibitor DUSP6 [56]. Notably, following treatment with 200 nM rotenone, PF-06447475 effectively reduced the Drp1 Ser616 phosphorylation level in mutant astrocytes but had no significant effect on wild-type astrocytes. In contrast, DUSP6 significantly decreased the Drp1 Ser616 phosphorylation levels in both wild-type and mutant astrocytes (Fig. 5a–h). This differential effect suggests distinct inhibition targets. DUSP6 may effectively reduce Drp1 Ser616 phosphorylation induced by both *LRRK2* G2019S mutation and rotenone treatment. Thus, we selected DUSP6 as the rescue approach for subsequent studies.

Our results revealed that in both the wild-type and the mutant coculture systems, treatment with DUSP6 (0.25 μg/mL) for 24 h significantly increased the colocalization of STX17 and mitochondria, improved mitochondrial transfer, and rescued the dendritic length of DA neurons (Fig. 6). Strikingly, DUSP6 did not have a significant protective effect on wild-type DA neurons, but did have a significant neuroprotective effect on mutant neurons. Such difference may be explained that in the mutant coculture system, *LRRK2* G2019S mutation and rotenone exposure collectively induce excessive phosphorylation, which results in a significantly higher Drp1 Ser616 phosphorylation level (Fig. 3f), more pronounced mitochondrial transfer defects (Fig. 2d–g) and, subsequently, more prominent dendritic shortening than in wild-type astrocytes (Fig. 2b, c). Therefore, our results demonstrate that in the PD model constructed from *LRRK2* G2019S-expressing iPSCs, DUSP6 (0.25 μg/mL, 24 h) has significant neuroprotective effects. However, the concentrations of DUSP6 in wild-type iPSC-based PD model require further exploration. These data provide an experimental foundation for our subsequent research and the exploration of new treatment strategies.

Overall, our study revealed that combined *LRRK2* G2019S mutation and rotenone lead to increased phosphorylation of Drp1 at Ser616, causing STX17 detachment from the outer mitochondrial membrane. This process results in reduced mitochondrial transfer efficiency and impairment of neural repair mediated by mitochondrial transfer. DUSP6 effectively reduces Drp1 activity, enhances the colocalization of STX17 and mitochondria, enhances mitochondrial transfer and neural repair functions, and has protective effects on both DA neurons carrying the *LRRK2* G2019S mutation and neurons exposed to the environmental toxin rotenone.

This study has two primary limitations. First, we analyzed iPSCs from the first PD patient in Chinese mainland carrying the *LRRK2* G2019S mutation [31]. This single-case design limits the generalizability of findings, as the observed effects may just reflect the features of the participant; thus, validation in larger cohorts is necessary. Second, the G2019S mutation is rare in the Han Chinese population‌, and whether its pathogenic mechanisms align with those in Western populations remains unclear. Future large-scale studies are essential to determine whether findings from this rare case in China can be extrapolated to Western populations. Moreover, other *LRRK2* mutations (e.g., *LRRK2* R1441G/C/H mutations) and other kinases with increased activity deserve further investigation and are crucial for assessing the generalizability of DUSP6 therapy in PD.

## Conclusions

In summary, this study proposes a novel perspective that mitochondrial transfer serves as an endogenous neurorepair mechanism, and its impairment may underlie the pathogenic mechanism of the *LRRK2* G2019S mutation. These findings elucidate the interplay between environmental factors and genetic background in PD and identify a new therapeutic target for both sporadic and *LRRK2* G2019S-mutant PD.

## Supplementary Information

Additional file 1. **Fig. S1**. Demographic information of PD patients. **Fig. S2**. Karyotype analysis and normal morphology, mycoplasma detection of iPSCs. **Fig. S3**. The LRRK2 G2019S mutation affects mitochondrial transfer rather than changes in quantity. **Fig. S4**. The LRRK2 G2019S mutation increases the phosphorylation level of Ser1292 rather than the transcriptional level of mRNA. **Fig.** **S5**. The activity of mitophagy did not change significantly in healthy and mutant astrocytes exposed to different concentrations of rotenone.

Additional file 2. All original, full-length gel and blot images.

## Data Availability

All data generated or analyzed during this study are included in this published article and its supplementary information files.

## Abbreviations

PD: Parkinson’s disease
iPSCs: Induced pluripotent stem cells
ACM: Astrocyte-conditioned medium
NSCs: Neural stem cells
TH: Tyrosine hydroxylas
Tuj1: Class III beta-tubulin
GFAP: Glial fibrillary acidic protein
PBMCs: Peripheral blood mononuclear cells
SNAP: Synaptic-associated protein family
VAMP: Vesicle-associated protein family
BDNF: Brain-derived neurotrophic factor
ROC: Ras of complex
Drp1: Dynamin-related protein 1
SNARE: Soluble N-ethylmaleimide-sensitive factor attachment receptor
