# Correction: *LRRK2* G2019S mutation contributes to mitochondrial transfer dysfunction in a Drp1-STX17-dependent manner

**DOI:** 10.1186/s40035-025-00533-1

**Published:** 2026-02-14

**Authors:** Mei Ding, Fen Wang, Lan‑Lan Jiang, Chao Ma, Yu‑Wan Qi, Jun‑Yi Liu, Juan Li, Mei‑Xia Wang, Hong Jin, Jin‑Ru Zhang, Cheng‑Jie Mao, Xiao‑Kang Li, Chun‑Feng Liu, Xiao‑Yu Cheng

**Affiliations:** 1 Department of Neurology and Clinical Research Center of Neurological Disease, The Second Affiliated Hospital of Soochow University, Suzhou, China; 2 Department of Neurology, Nantong First People’s Hospital, Nantong, China; 3 Jiangsu Key Laboratory of Drug Discovery and Translational Research for Brain Diseases, Institute of Neuroscience, Soochow University, Suzhou, China; 4 Department of Neurology, Xiongan Xuanwu Hospital, Xiongan, China; 5 Department of Neurology, The Fourth Affiliated Hospital of Soochow University, Suzhou, China; 6 School of Pharmacy, Ningxia Medical University, Yinchuan, China; 7 Department of Neurology, Suzhou Municipal Hospital, Suzhou Hospital Affiliated to Nanjing Medical University, Suzhou, China; 8 Laboratory of Transplantation Immunology, National Research Institute for Child Health and Development, Tokyo, Japan

**Correction:** ***Transl Neurodegener*** **14, 64 (2025). **10.1186/s40035-025-00525-1

During daily data collation and inspection, the authors found that Figs. 2 and 3 in recently published article [1] contained mistakes during the figure editing and version uploading process, which ultimately led to the incorrect version being online due to carelessness.

In the third row (AS^*LRRK2*^ ^G2019S^ + Neuron ^*LRRK2*^ ^G2019S^ group) of Fig. 2b, the fluorescence images for the **control group** and the **Rot 50 nM** group were incorrect. In the third row of Fig. 3i, there was a typo regarding the Western blots bands (**STX17**). In the third row (**Healthy, 100 nM Rot group**) of Fig. 3l, the fluorescence image was incorrect.

Figure 2 has been corrected from:

**Figure 1:**
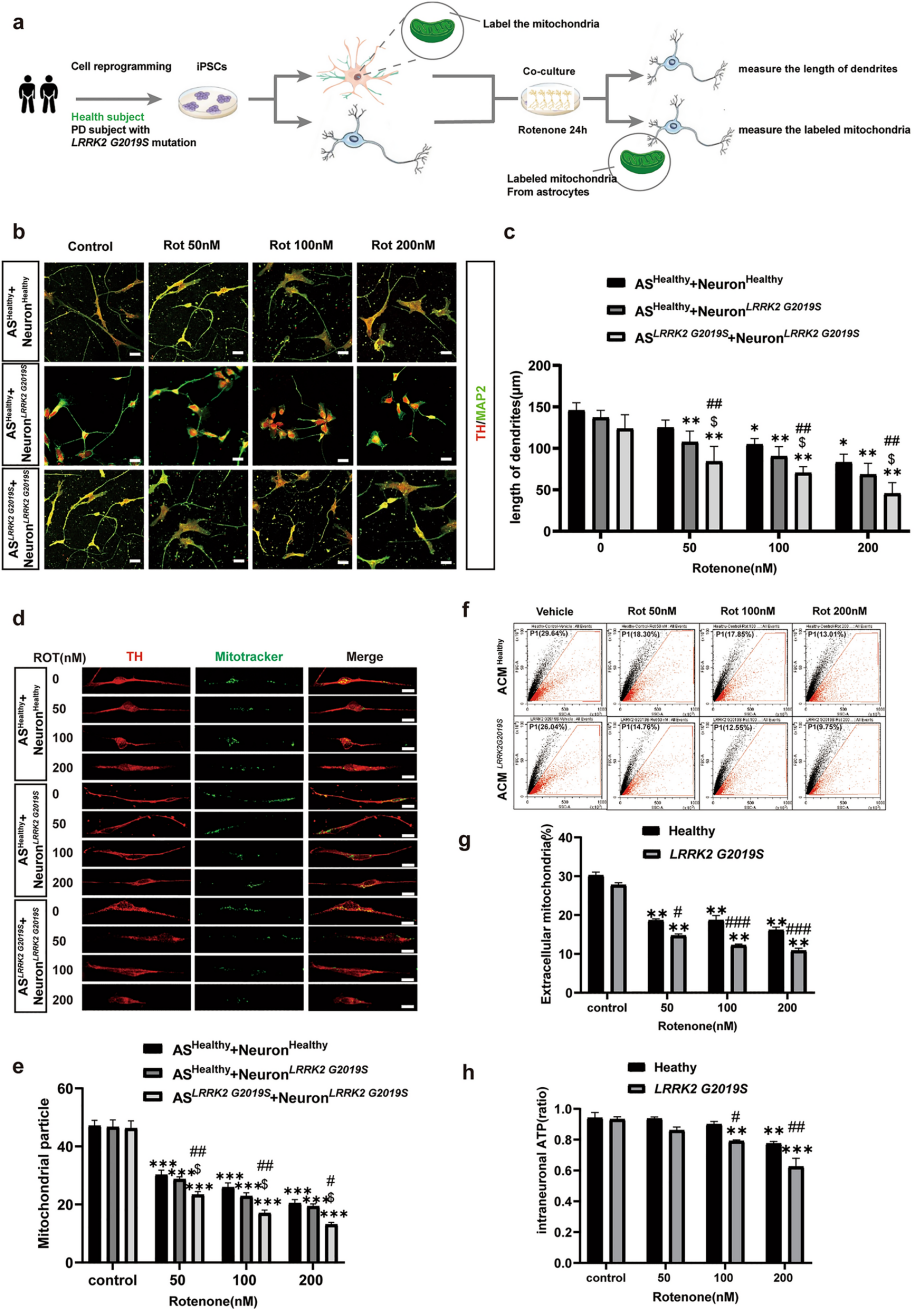

To:

**Figure 2:**
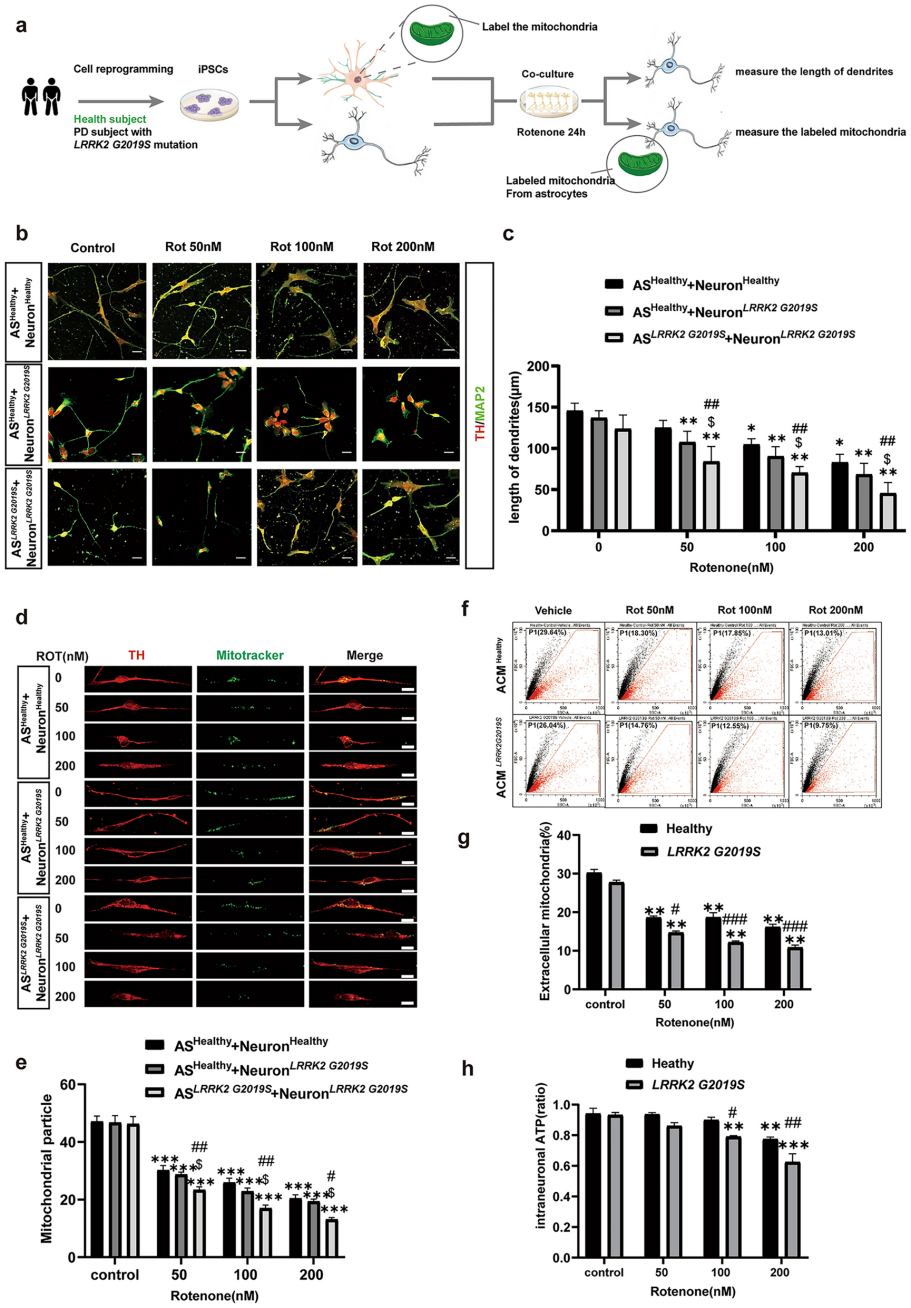

Figure 3 has been corrected from:

**Figure 3:**
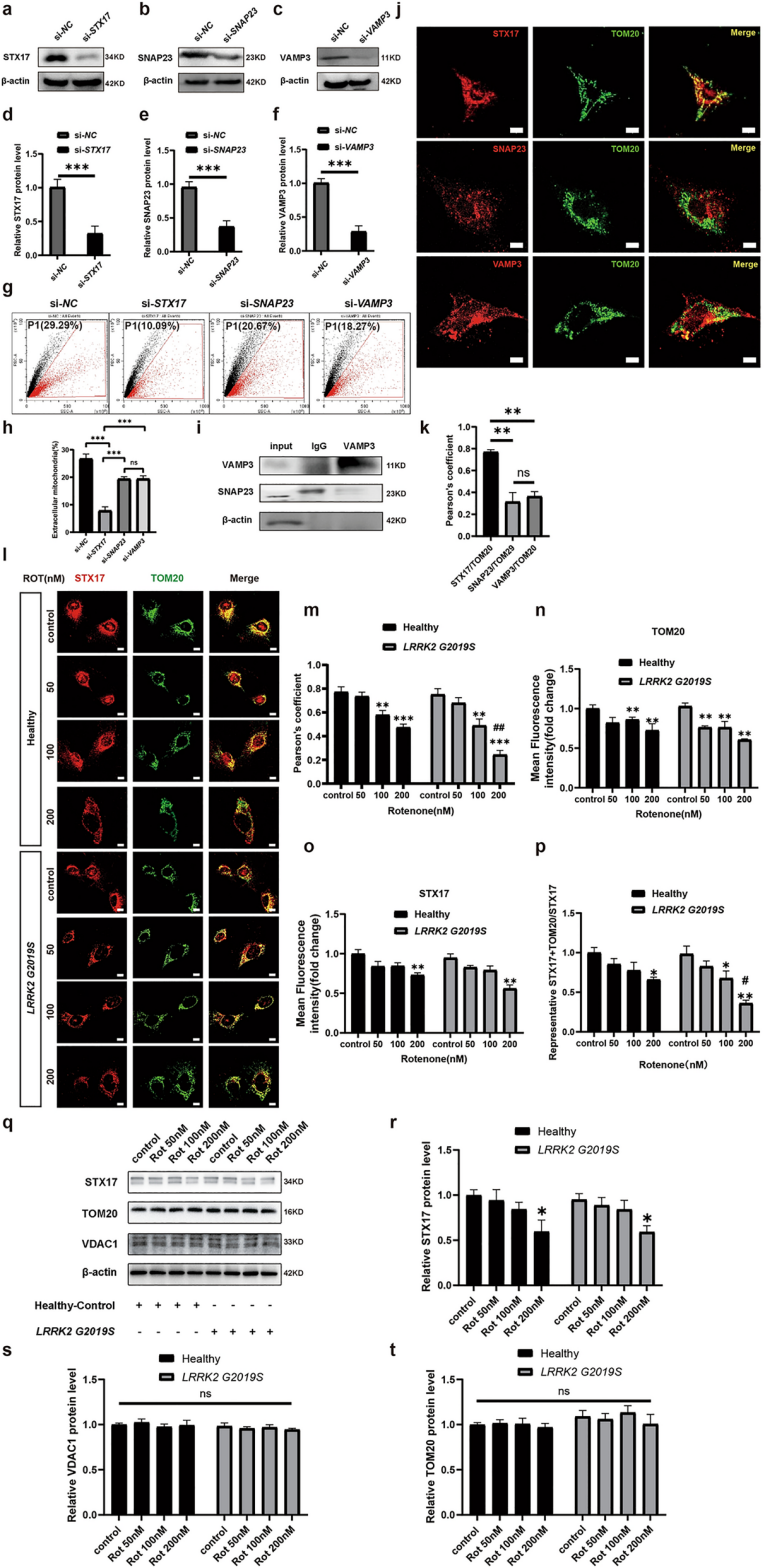

To:

**Figure 4:**
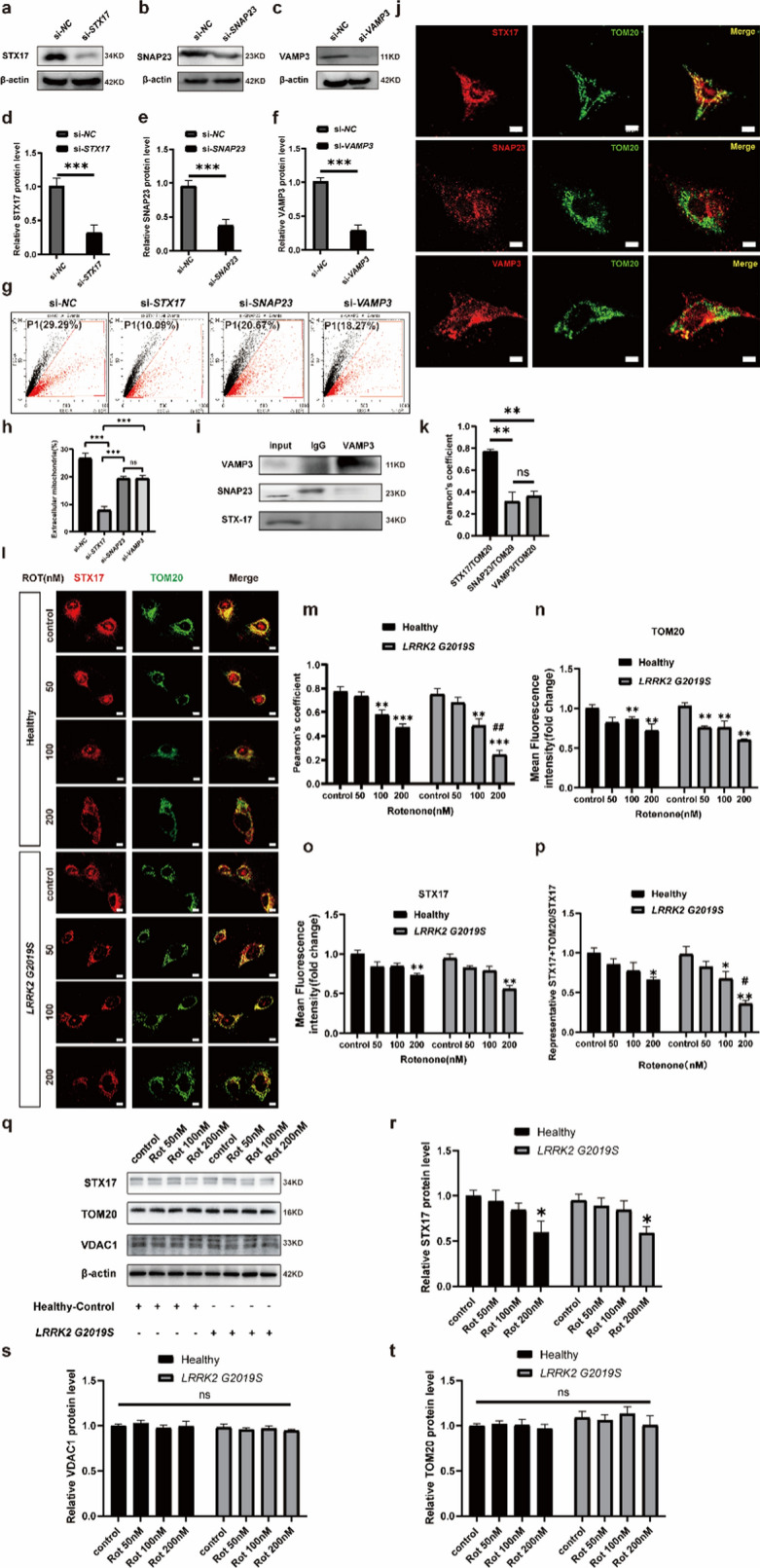

The underlying experimental data and their descriptions in the Results section and figure legends remain accurate and unchanged.

The original article [1] has been corrected.
