# Supplementary material for: LRRK2 G2019S mutation contributes to mitochondrial transfer dysfunction in a Drp1-STX17-dependent manner
Source: Transl Neurodegener. 2025 Dec 8;14:64. doi: 10.1186/s40035-025-00525-1 (PMC12683823; doi:10.1186/s40035-025-00525-1)
Supplement: Supplementary file 1 — Additional file 1. Fig. S1. Demographic information of PD patients. Fig. S2. Karyotype analysis and normal morphology, mycoplasma detection of iPSCs. Fig. S3. The LRRK2 G2019S mutation affects mitochondrial transfer rather than changes in quantity. Fig. S4. The LRRK2 G2019S mutation increases the phosphorylation level of Ser1292 rather than the transcriptional level of mRNA. Fig. S5. The activity of mitophagy did not change significantly in healthy and mutant astrocytes exposed to different concentrations of rotenone. [file 40035_2025_525_MOESM1_ESM.docx]

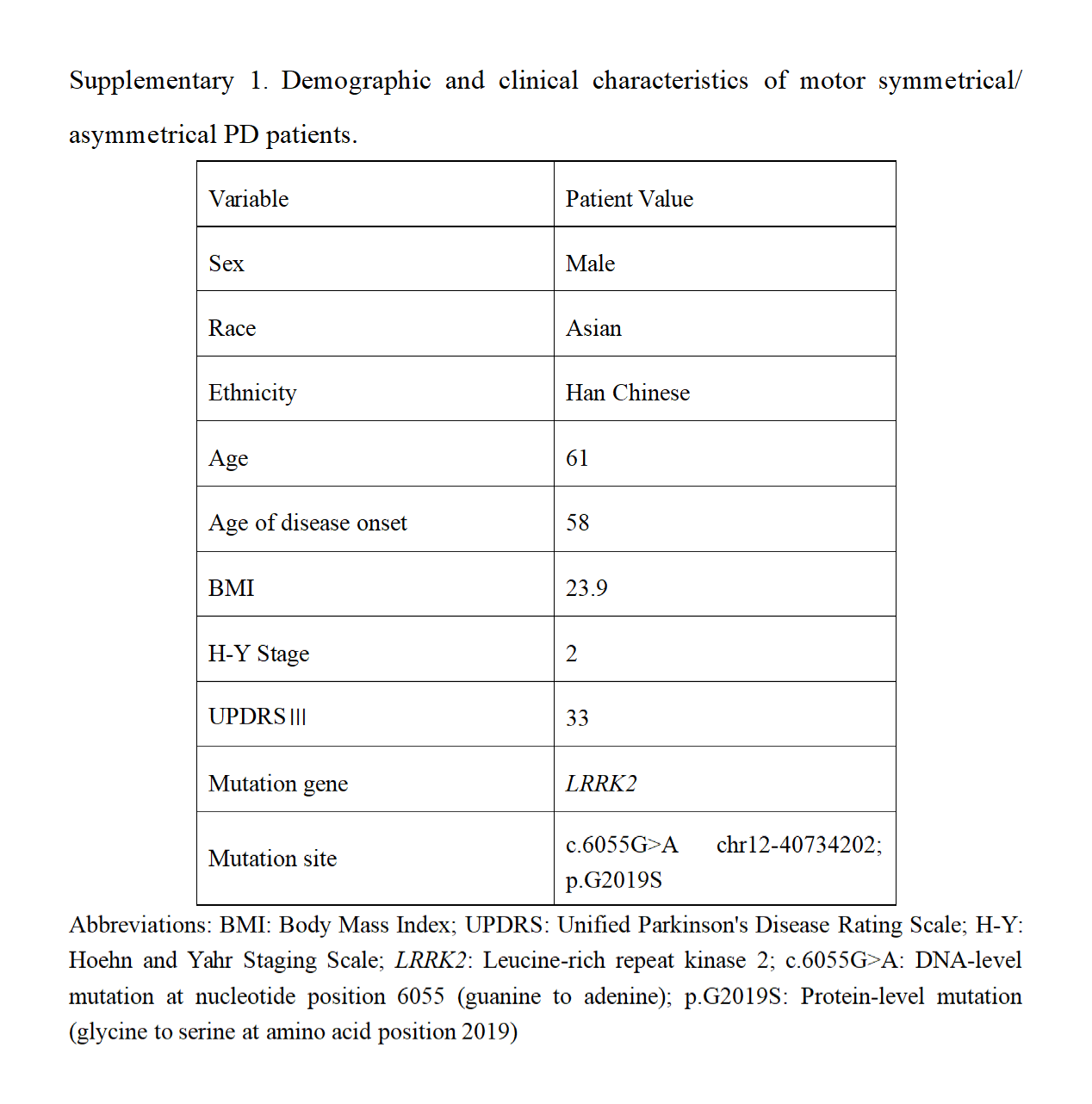
**Fig. S1. Demographic Information of PD Patients.** The data included sex, race, ethnicity, age, age of disease onset, BMI, H-Y stage, UPDRS III score, mutation gene status, and mutation site.


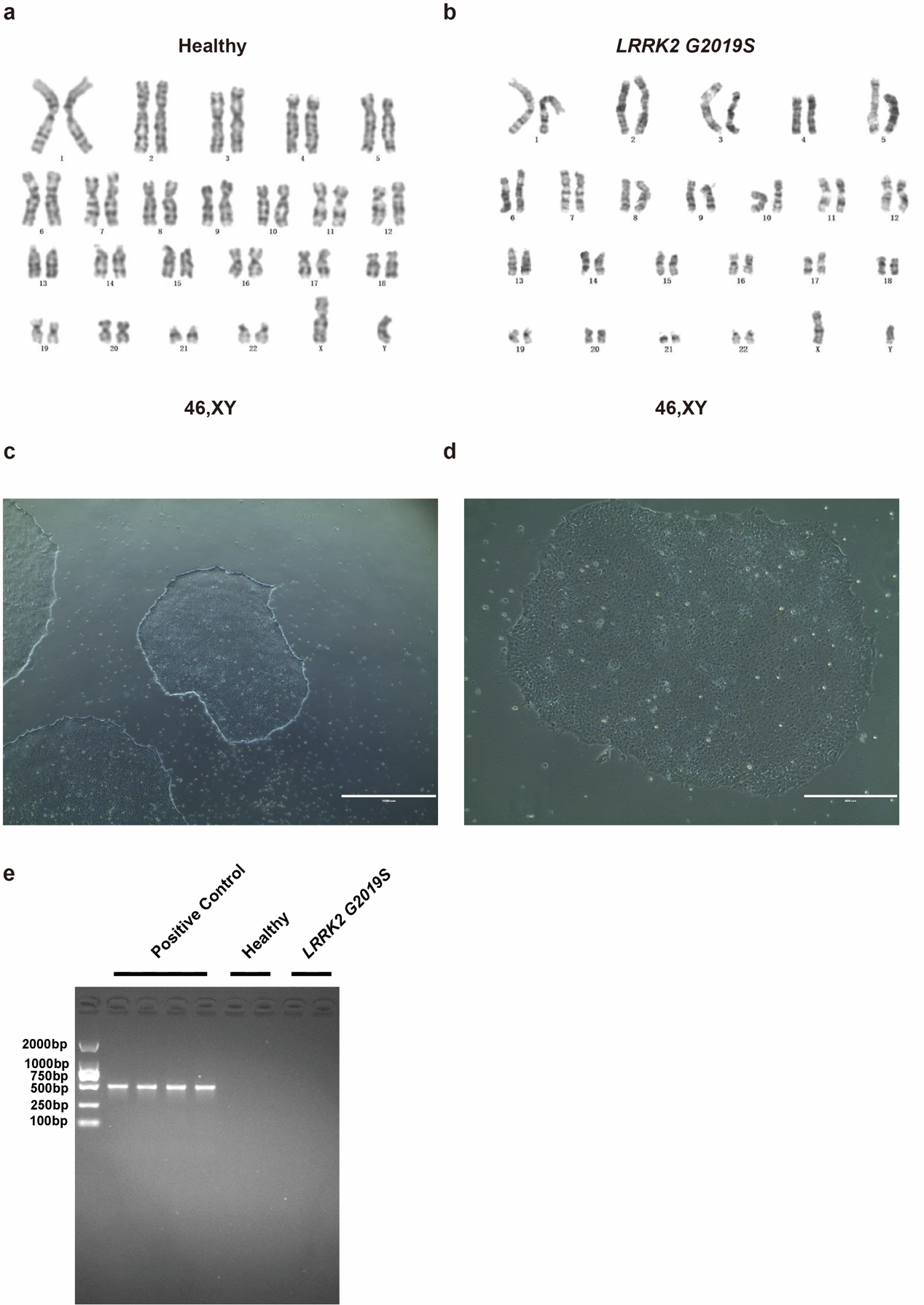


**Fig. S2.** Karyotype analysis and normal morphology, mycoplasma detection of iPSCs. **a-b** Karyotyping of iPSCs derived from the healthy volunteer and the PD patient carrying the *LRRK2* G2019S mutation. **c-d** Typical hiPSC colonies were screened by light microscopy. **e** Mycoplasma testing of the positive control, healthy iPSCs, and *LRRK2* G2019S iPSCs. The scale bars represent 1000 μm in (**c**) and 400μm in (**d**).


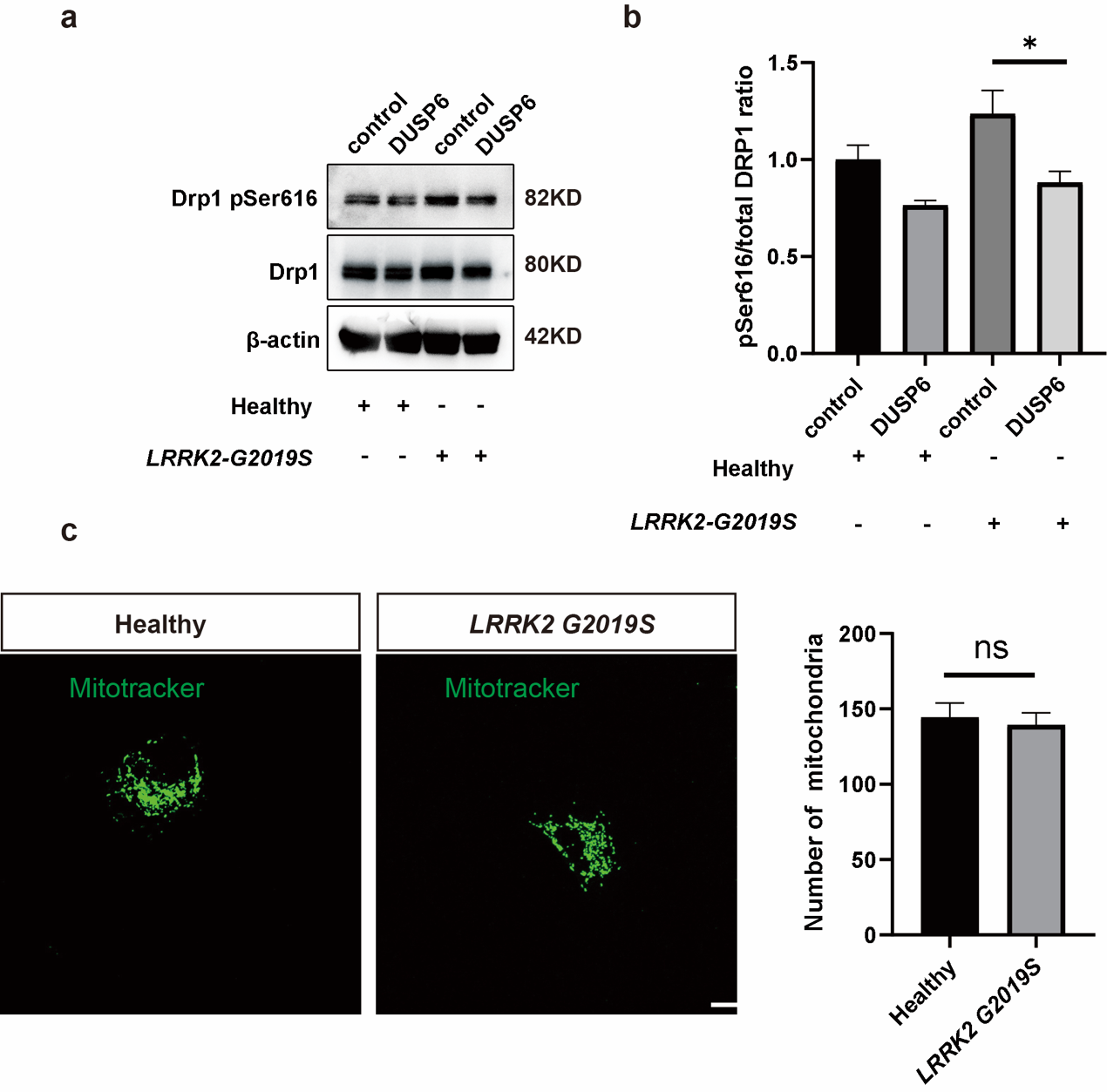


**Fig. S3.** The *LRRK2* G2019S mutation affects mitochondrial transfer rather than changes in quantity. **a** Western blots showing the amount of total Drp1 and Drp1 pSer616 with or without DUSP6 (0.25 μg/μL, 24 h) treatment in wild-type and *LRRK2* G2019S astrocytes. **b** Quantification of the protein levels of Drp1 and Drp1 pSer616 in the experiments in **a**. **c** Immunostaining showing astrocyte mitochondria labeled with MitoTracker (green) for 30 minutes in wild-type and *LRRK2* G2019S astrocytes. **d** Quantification of the astrocytic mitochondria in wild-type and *LRRK2* G2019S astrocytes. Data information: Three independent experiments were performed (*n*=3). Data in (**b,d**); * *P*< 0.01. Statistical significance was determined by one-way ANOVA followed by Tukey's HSD test‌ in **b** and by t test analysis in **d**. The results are presented as the mean ± SEM. The scale bars in all panels represent 20 μm.


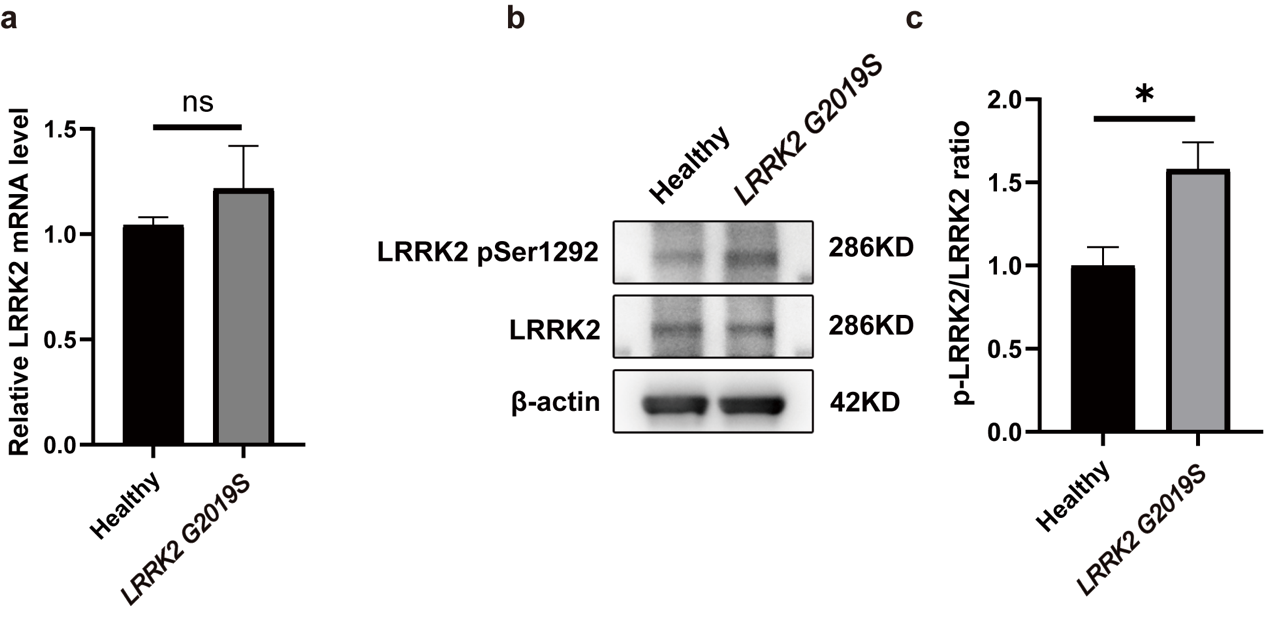


**Fig. S4.** The *LRRK2* G2019S mutation increases the phosphorylation level of Ser1292 rather than the transcriptional level of mRNA. **a** Quantitative PCR analysis showing the expression levels of *LRRK2* mRNA in healthy and *LRRK2* G2019S astrocytes. **b** Western blots showing the amount of LRRK2 and LRRK2 pSer1292 in healthy and *LRRK2* G2019S astrocytes. **c** Quantification of the ratio of LRRK2 pSer1292 to total LRRK2. Data information: Three independent experiments were performed (*n*=3). Statistical significance was determined by a t test. The results are presented as the mean ± SEM.


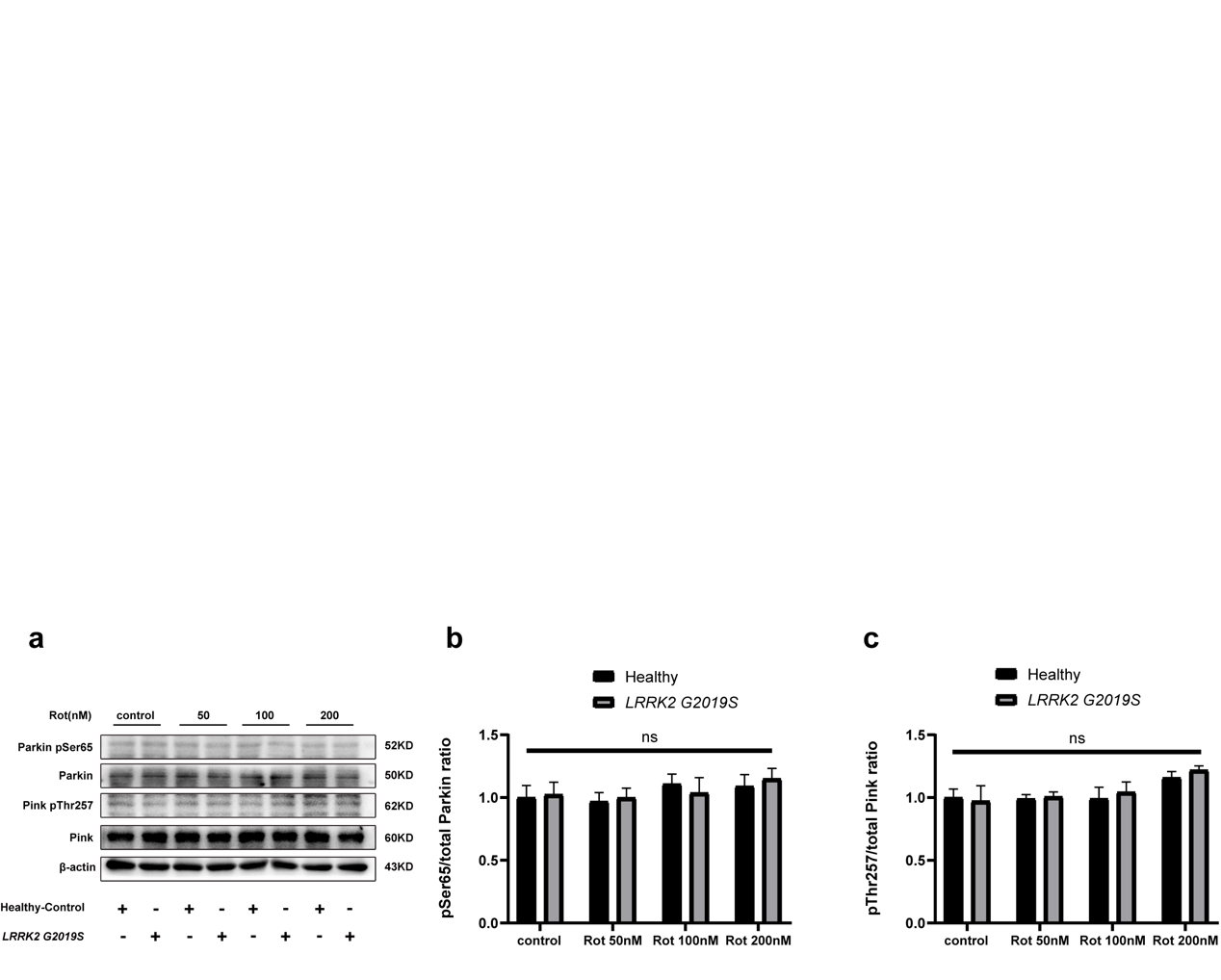


**Fig. S5.** The activity of mitophagy did not change significantly in healthy and mutant astrocytes exposed to different concentrations of rotenone. **a** Western blots showing the levels of Parkin, Parkin pSer65, Pink, and Pink pThr257 in healthy and mutated astrocytes treated with different rotenone doses. **b-c** Quantification of the ratio of Parkin pSer65 to total Parkin and Pink pThr257 to Pink. Data information: Three independent experiments were performed (*n*=3). Statistical significance was determined by two-way ANOVA followed by Tukey's HSD test‌. The results are presented as the mean ± SEM.
