# Supplementary material for: LRRK2 G2019S mutation contributes to mitochondrial transfer dysfunction in a Drp1-STX17-dependent manner
Source: Transl Neurodegener. 2025 Dec 8;14:64. doi: 10.1186/s40035-025-00525-1 (PMC12683823; doi:10.1186/s40035-025-00525-1)

**Raw data**

**Figure 1 C GFAP+GAPDH**


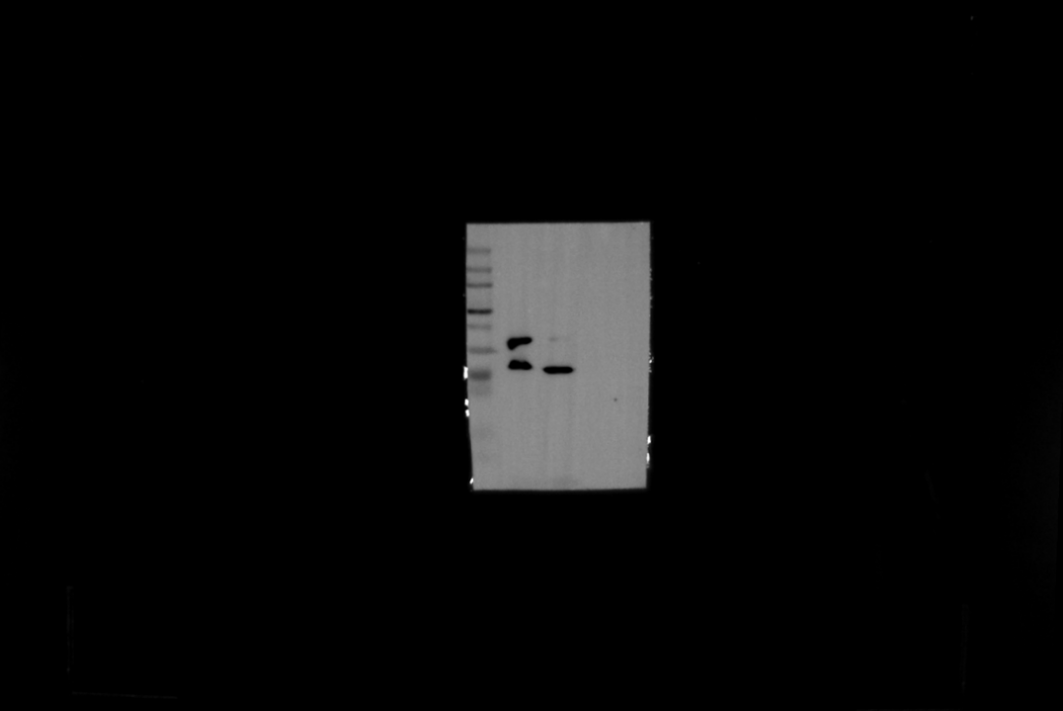


**Figure 1 C TH+β-actin**


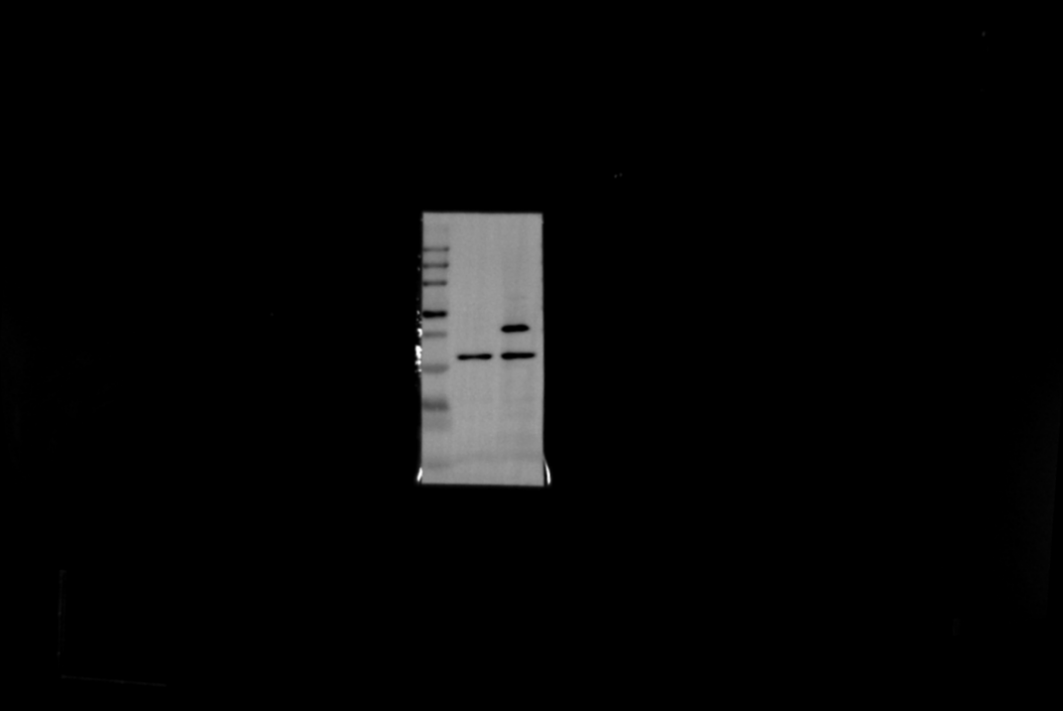


**Figure3A β-ACTIN**


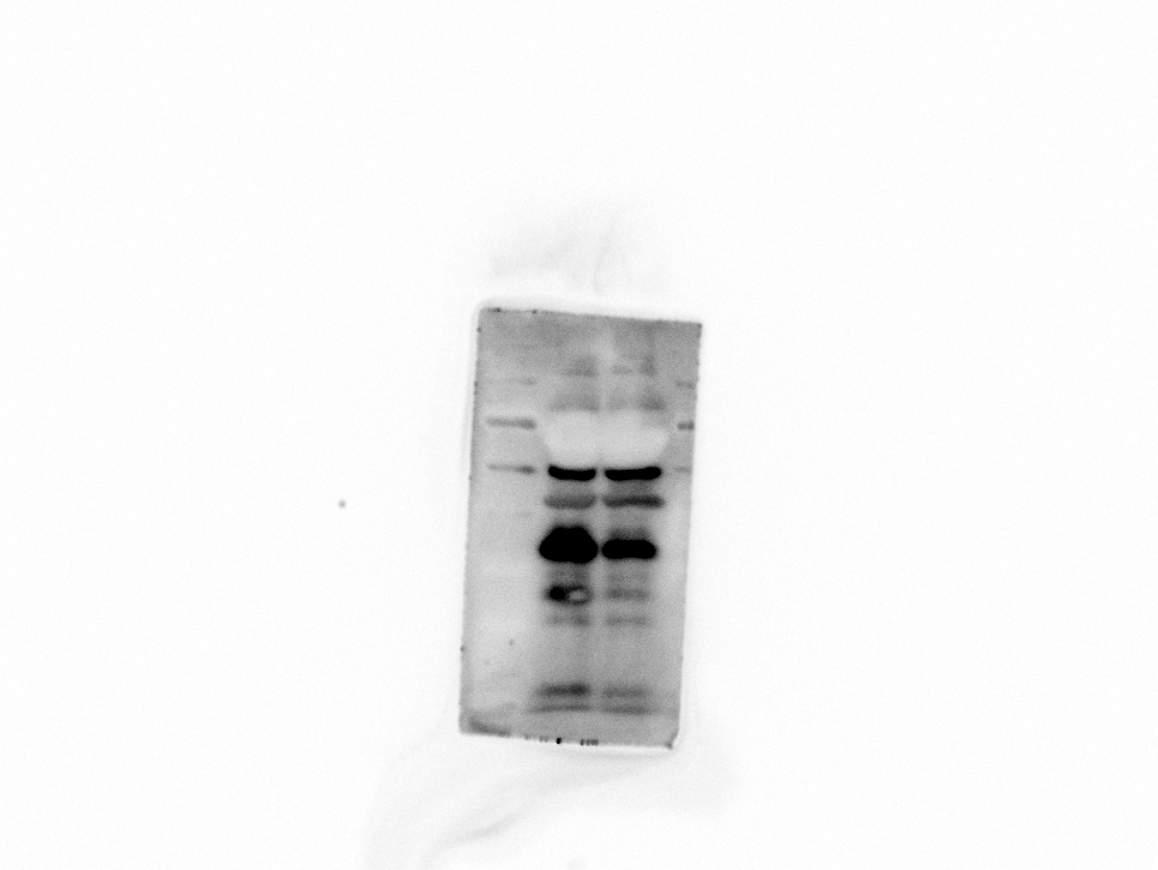


**Figure3A STX17**


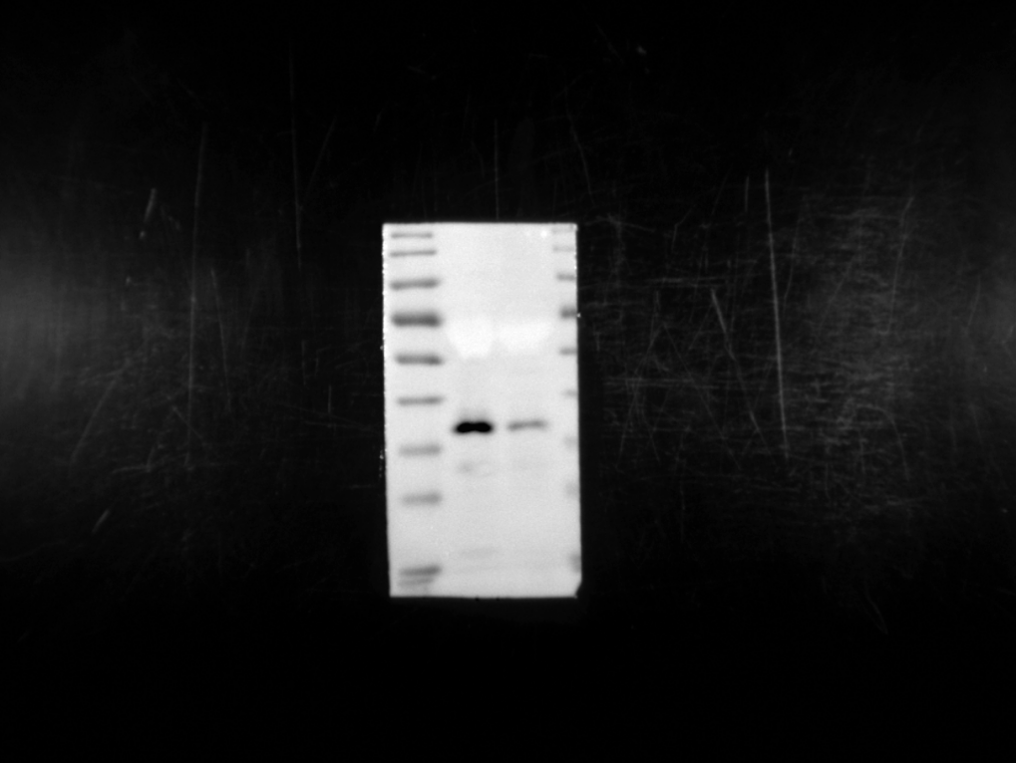


**Figure3B SNAP23**


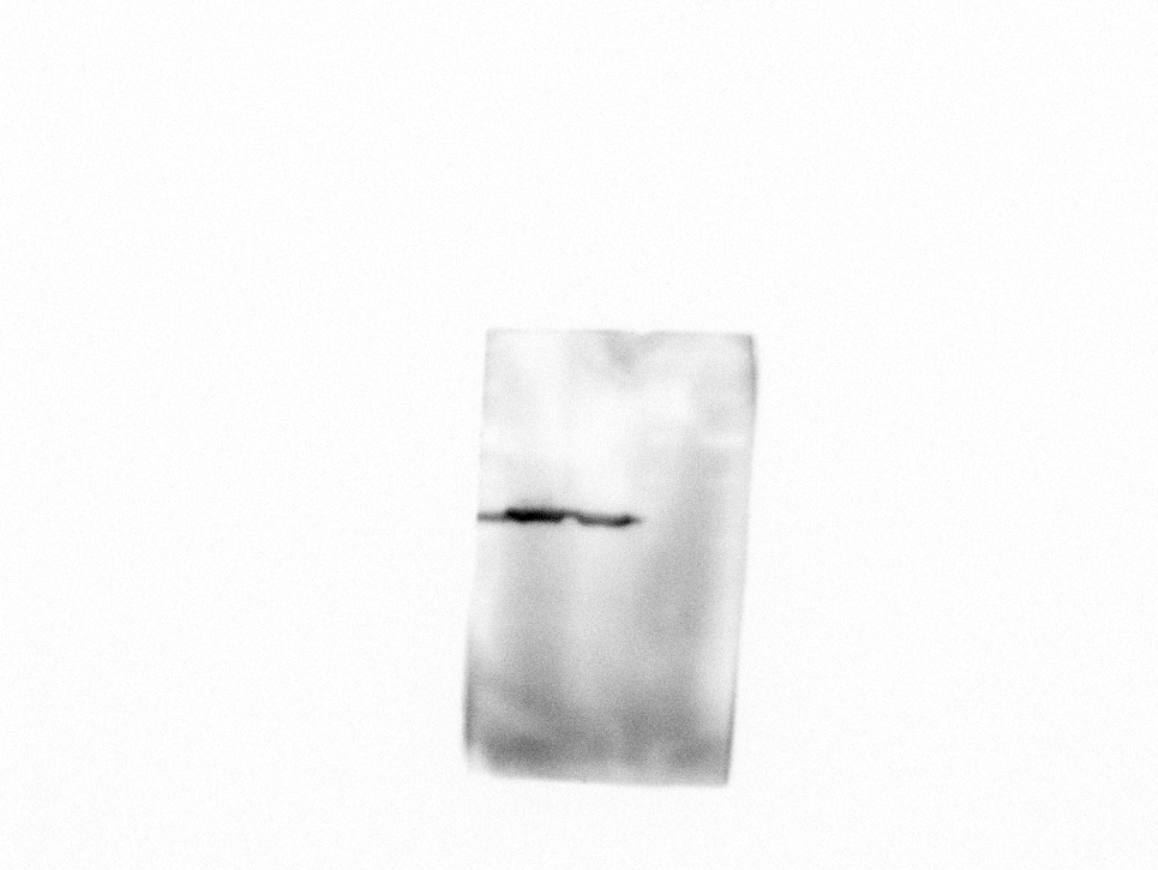


**Figure3B β-ACTIN**

**
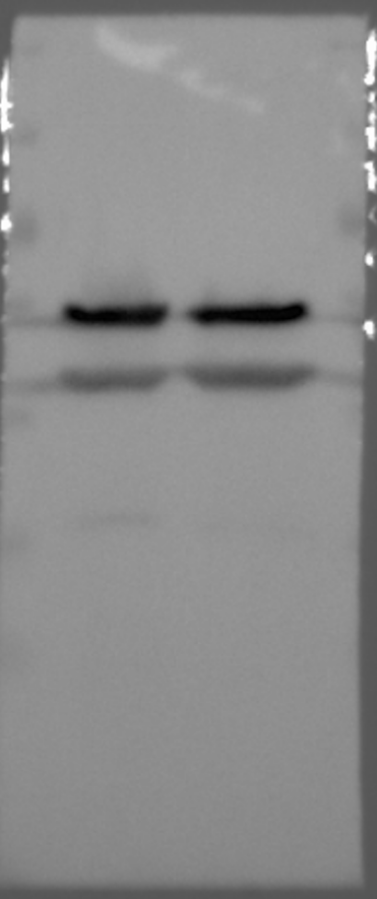
**

**Figure3C β-actin**


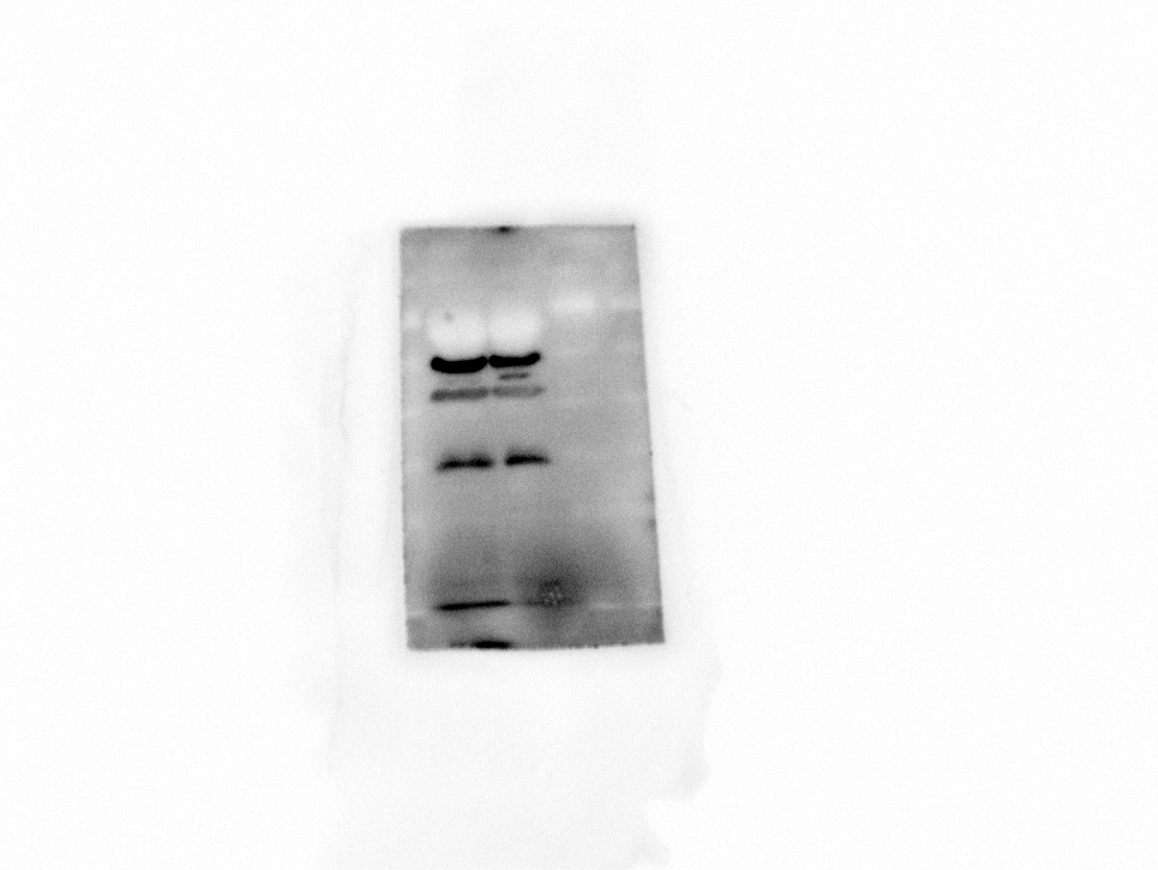


**Figure 3C VAMP3**


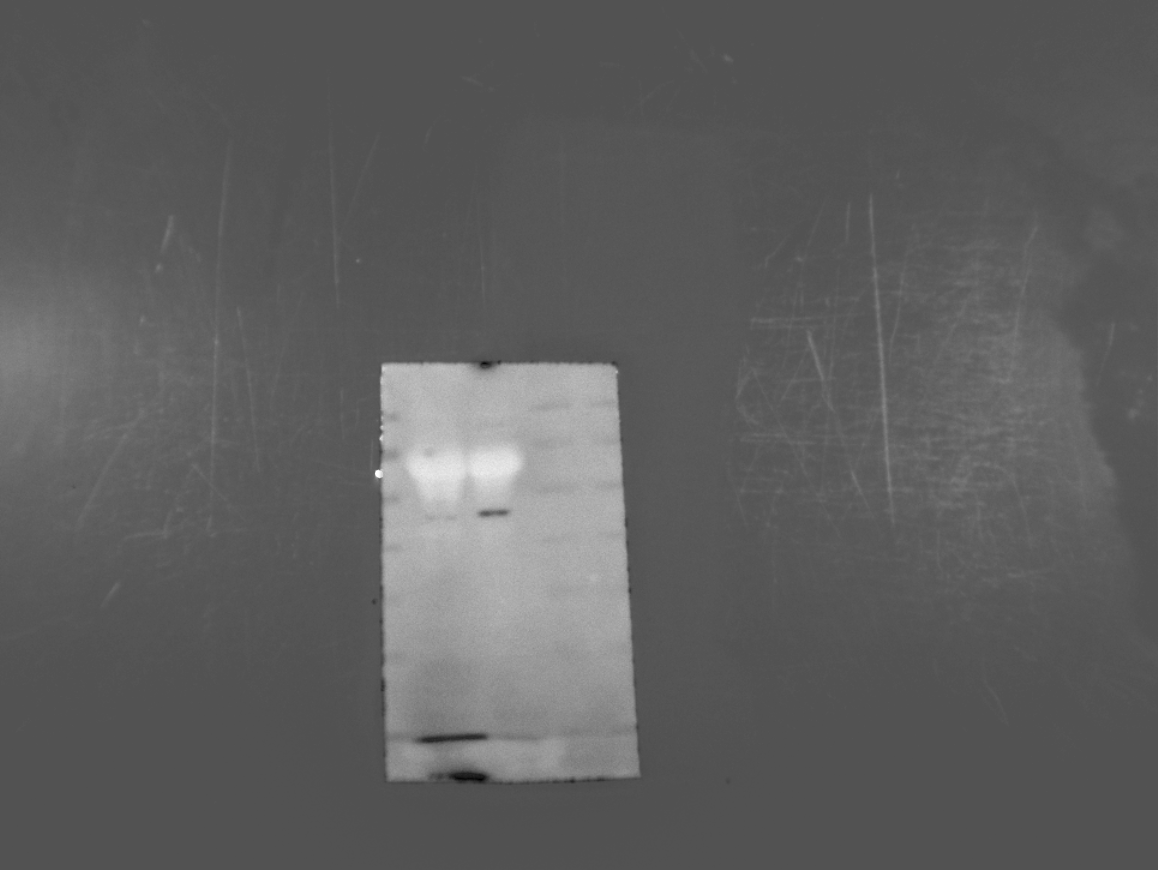


**Figure3I VAMP3**


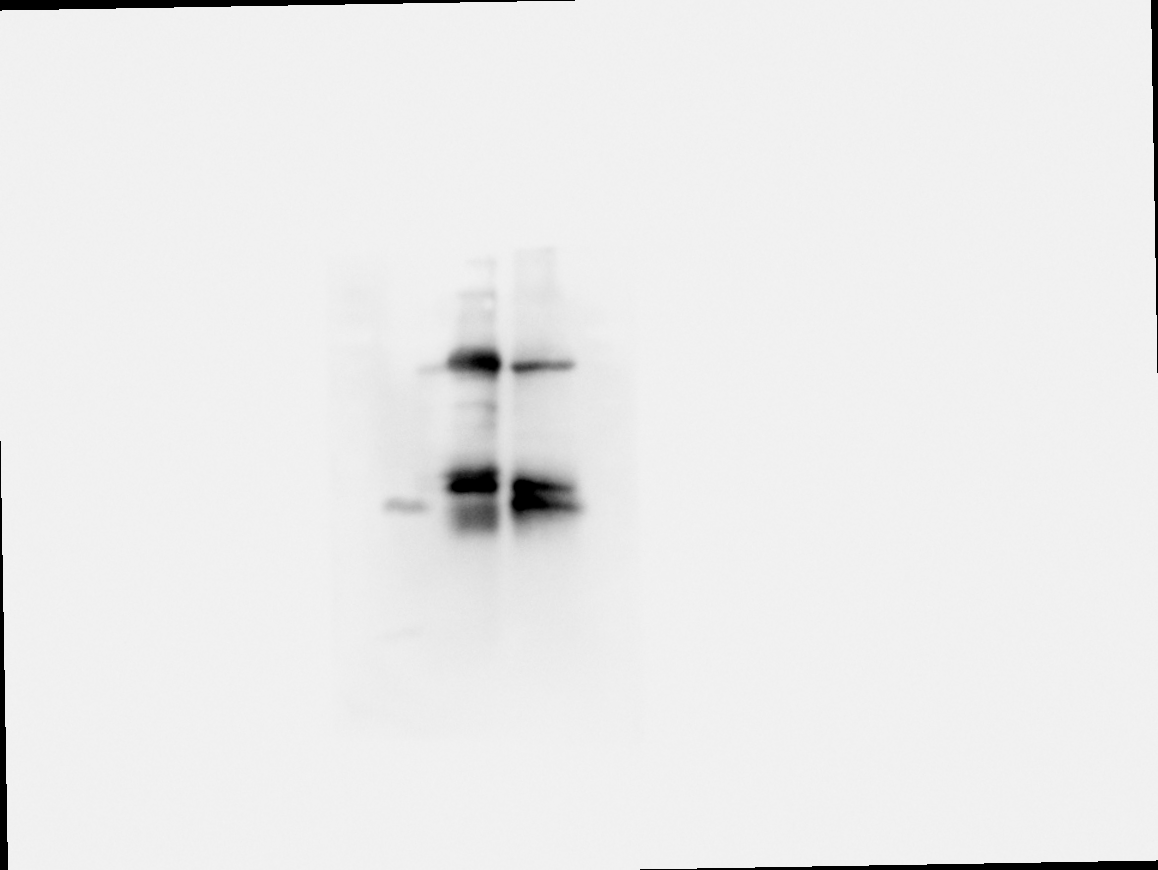


**Figure3I SANP23**


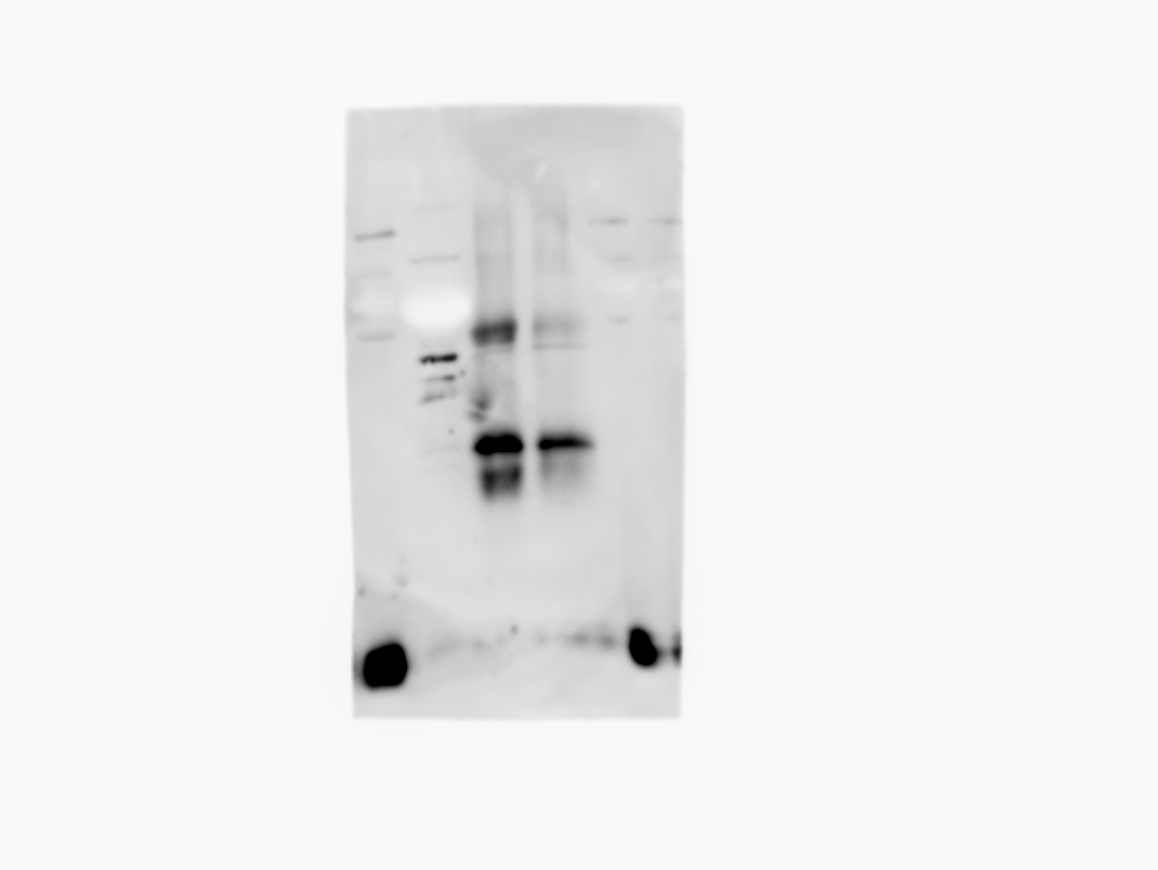


**Figure3I STX-17**


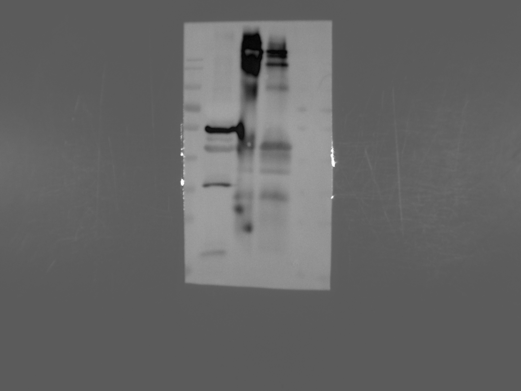


**Figure3Q-STX17**


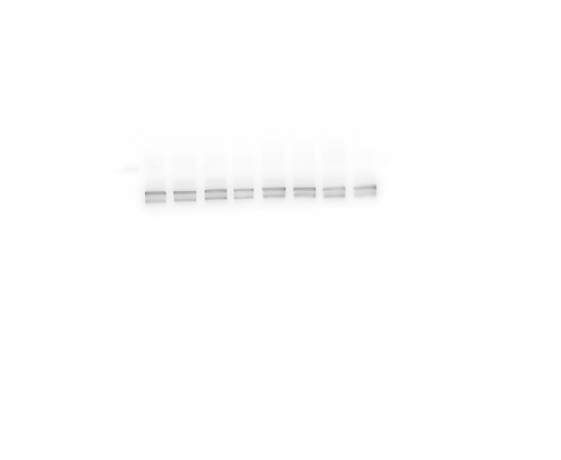


**Figure3Q-TOM20**


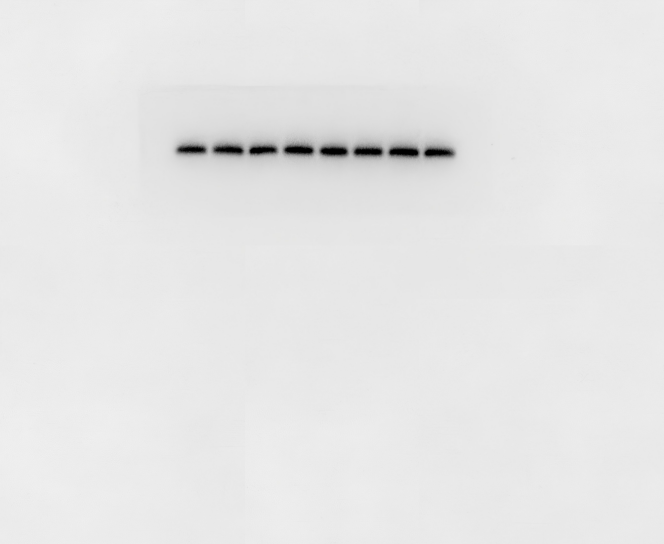


**Figure3Q-VDAC1**


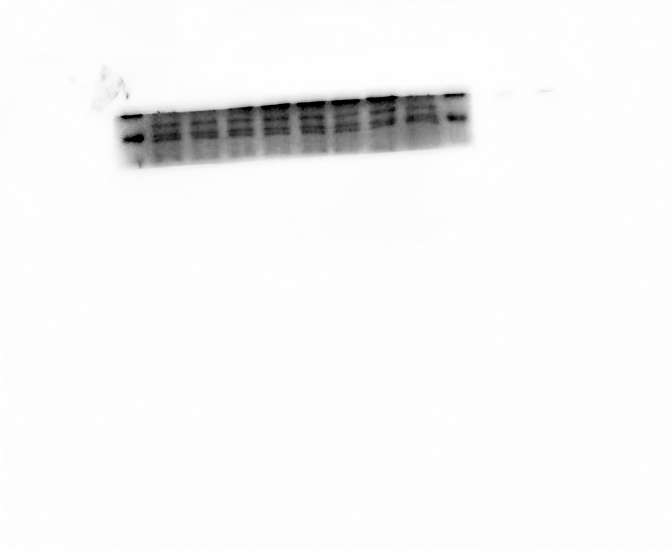


**Figure3Q-β-ACTIN**


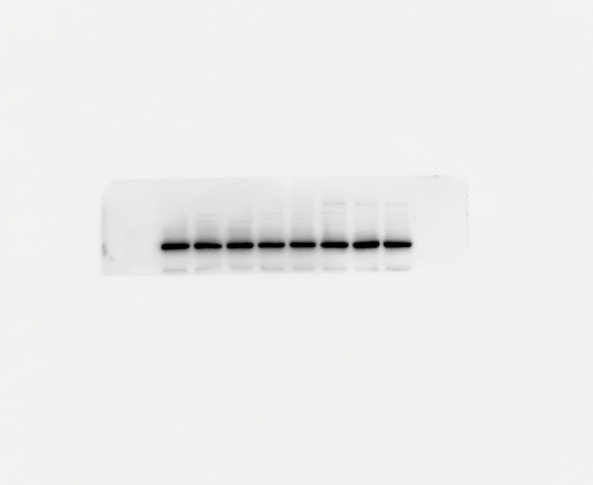


**Figure4A actin**


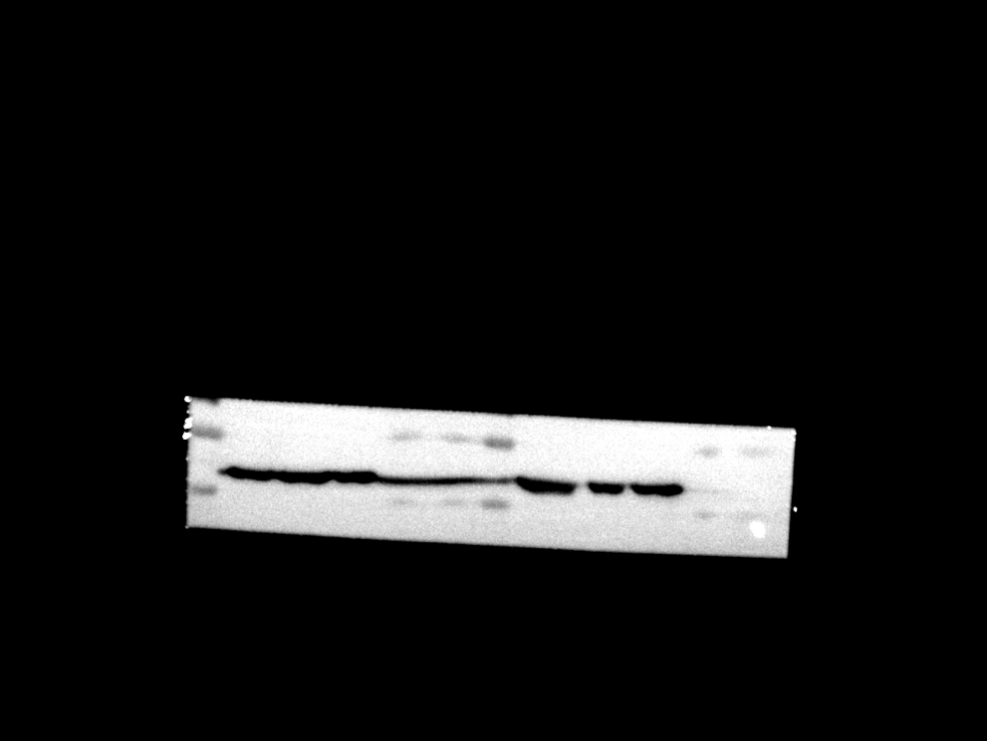


**Figure4A DRP1**


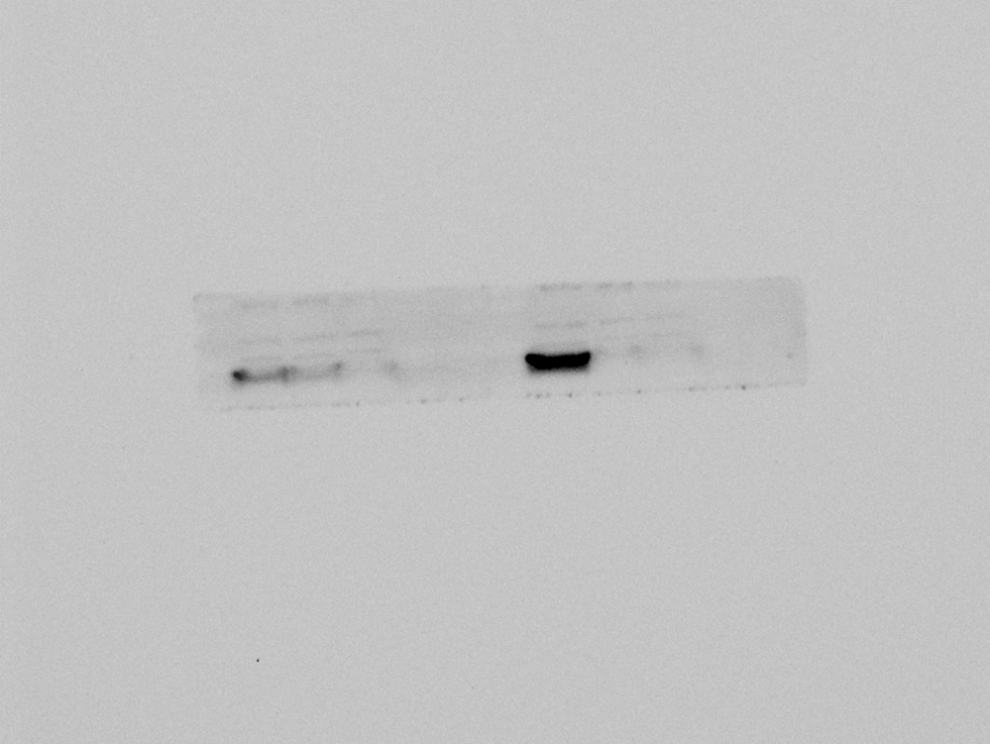


**Figure4 E IB DRP1**

**
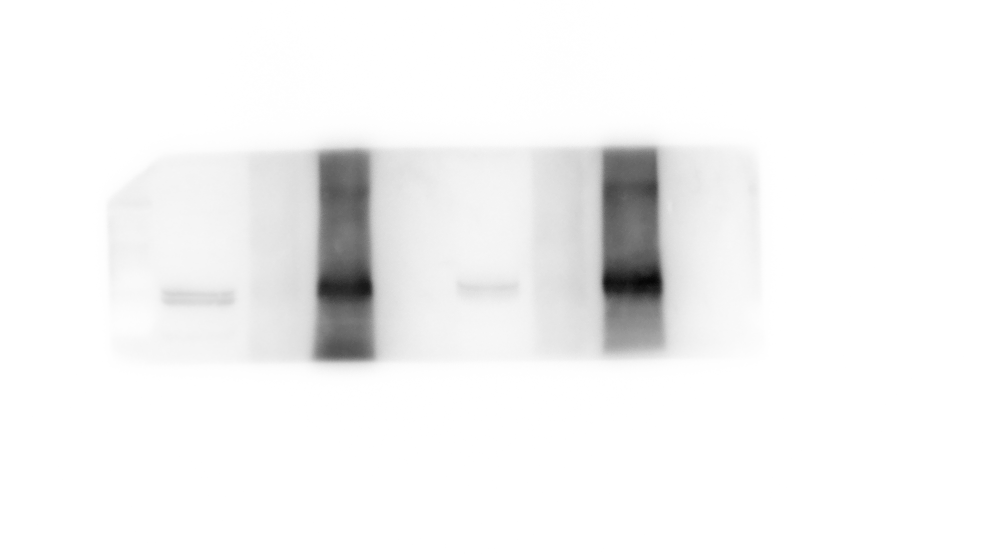
**

**Figure4 E IB STX17**

**
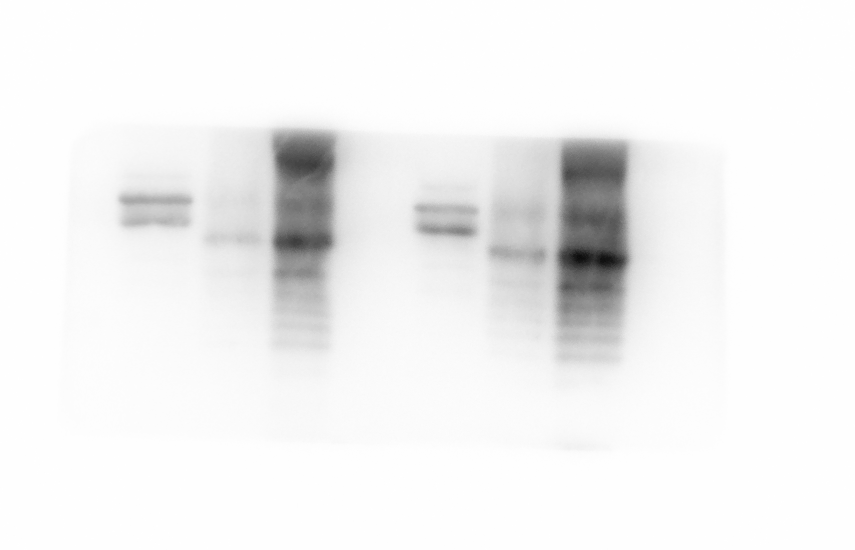
**

**Figure 4 F DRP1 / actin**


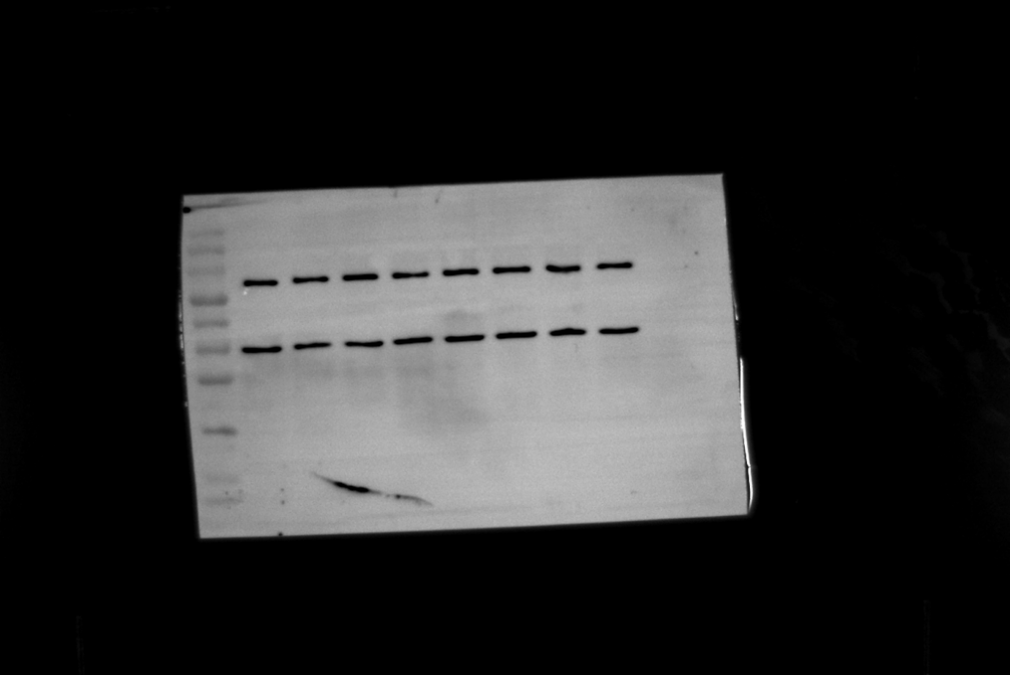


**Figure 4 F p616-DRP1**


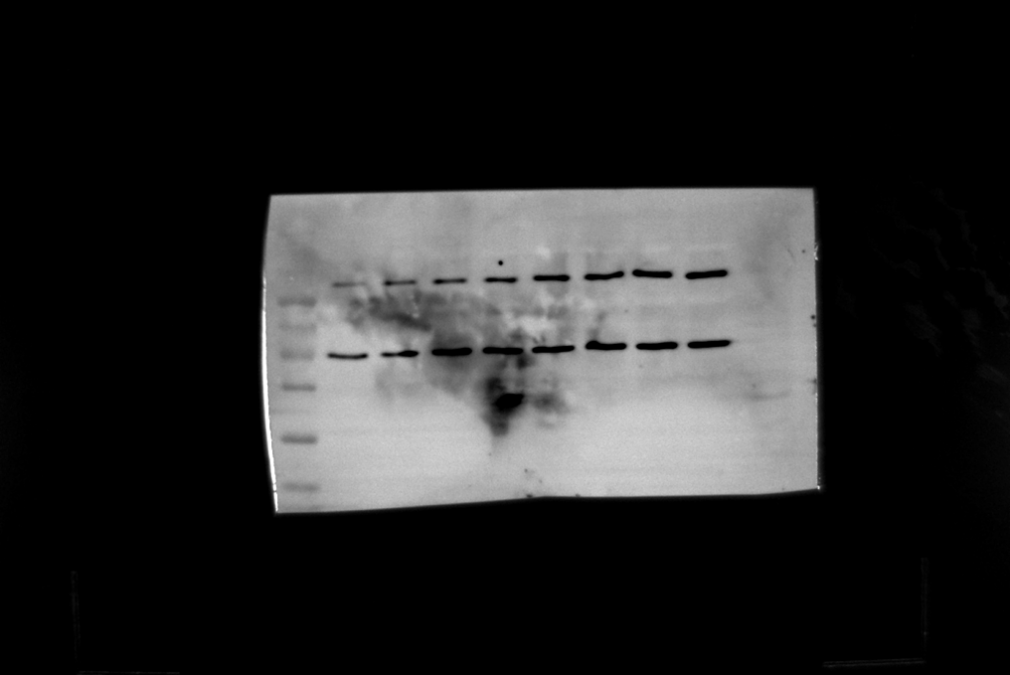


**Figure5A actin**


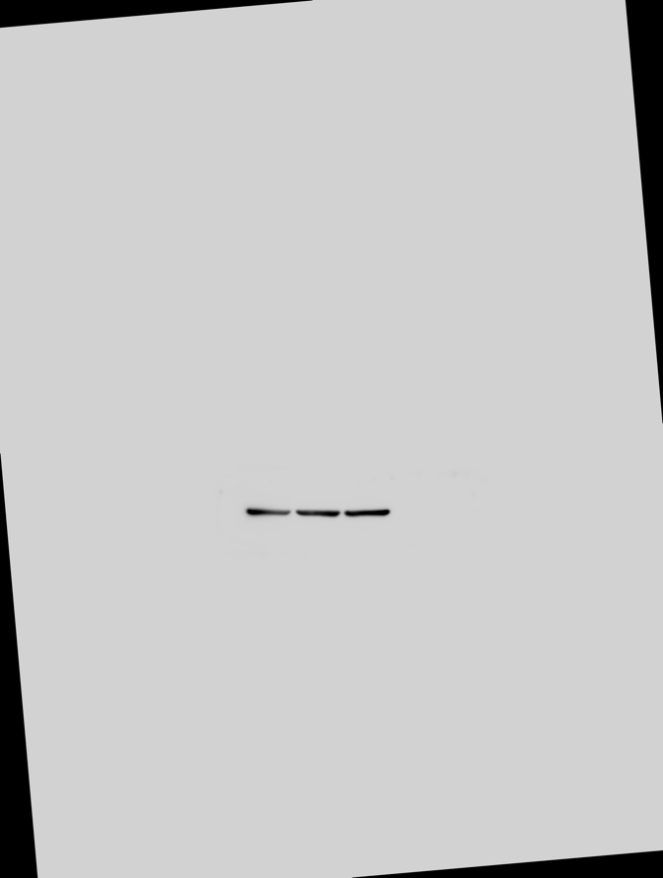


**Figure5A drp1**


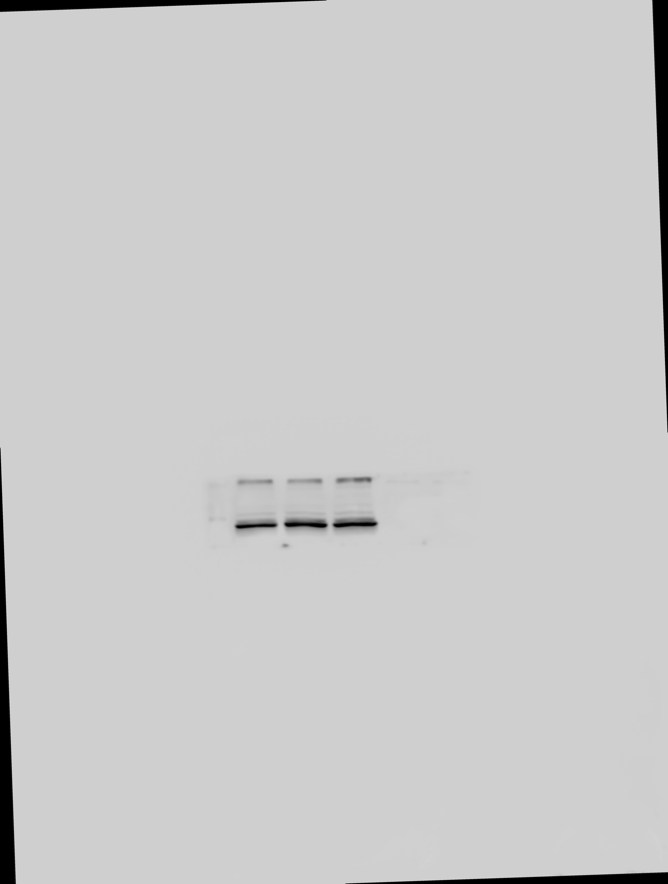


**Figure5A P-drp1**


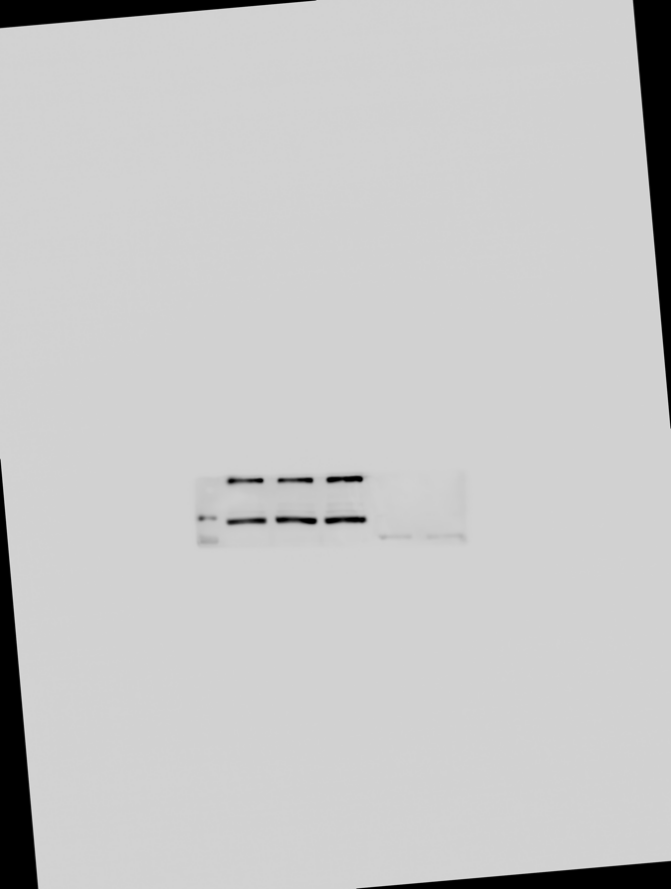


**Figure5B actin**


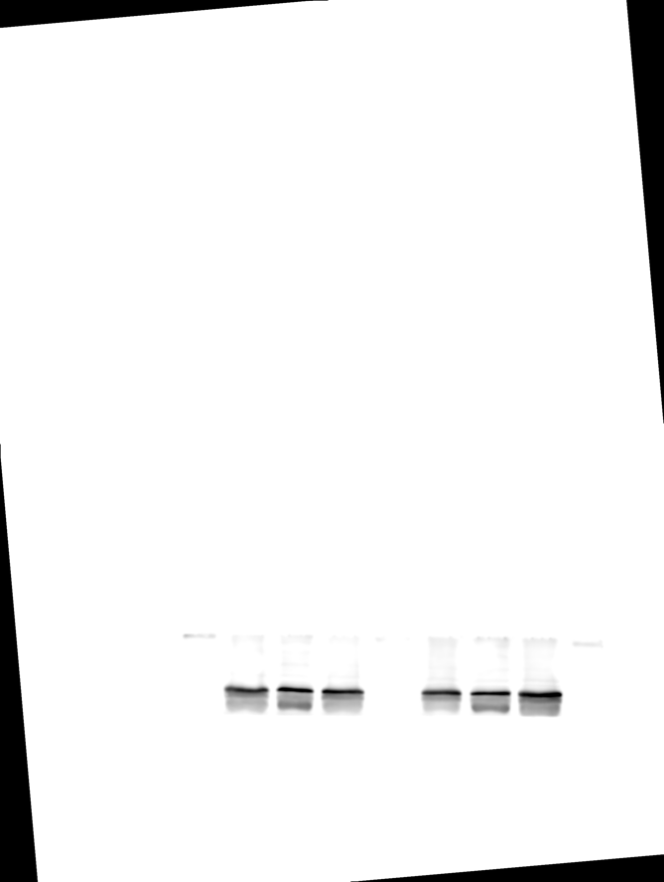


**Figure5B drp1**


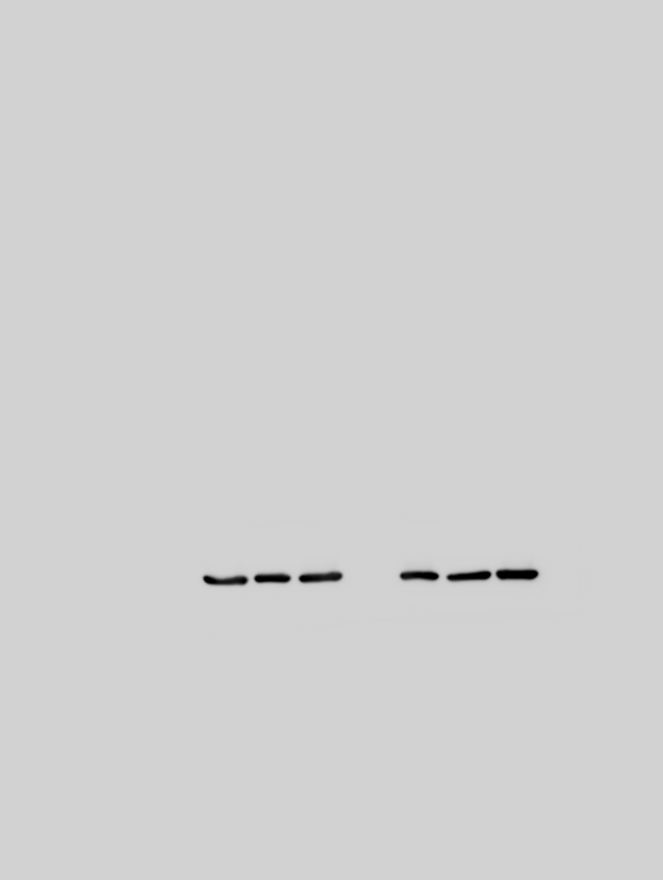


**Figure5B p-drp1**


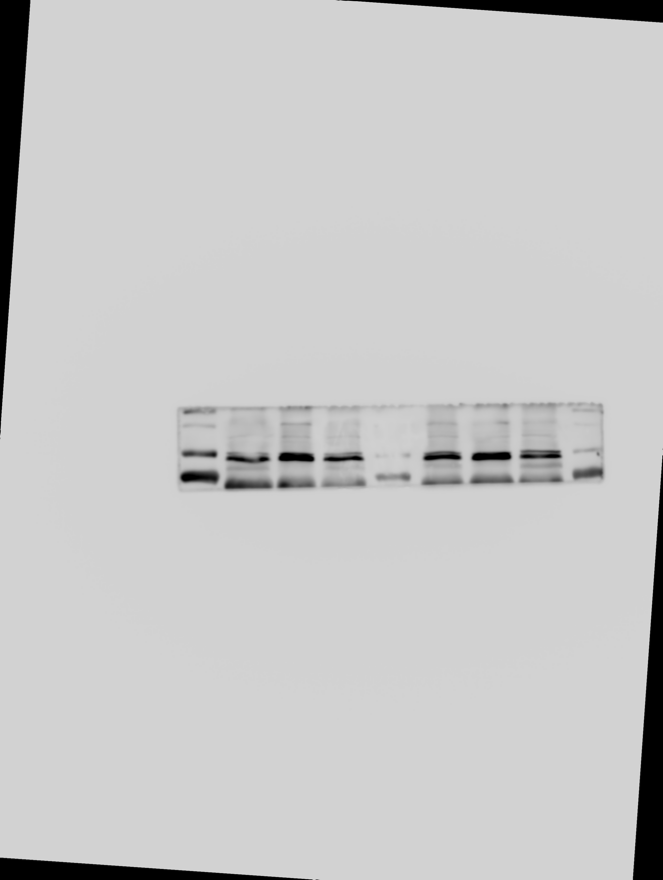


**Figure5C actin**


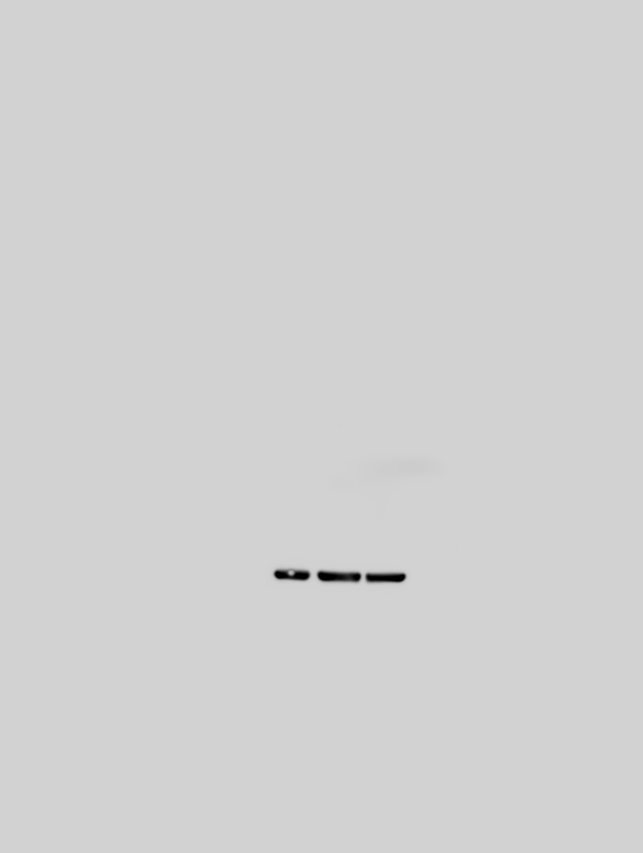


**Figure5C -drp1**


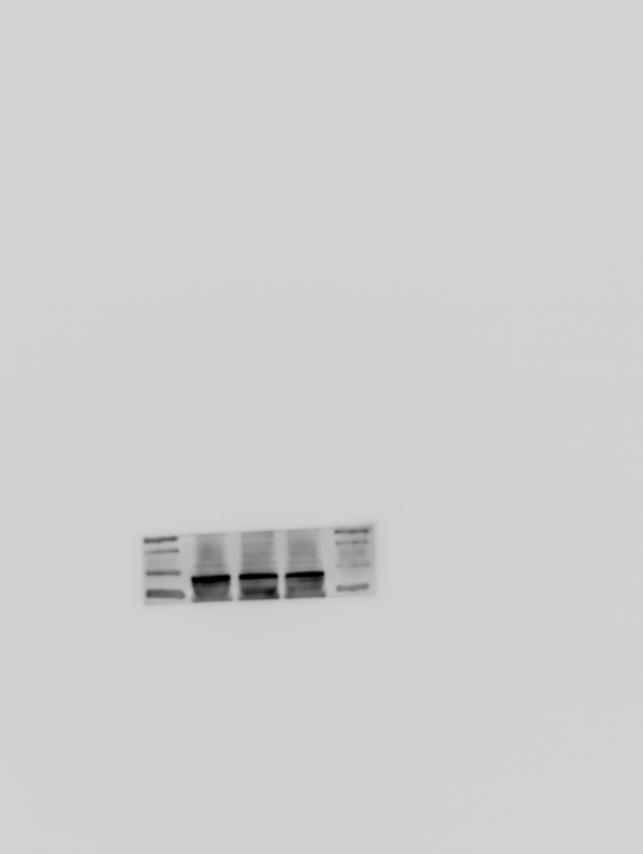


**Figure5C p-DRP1**


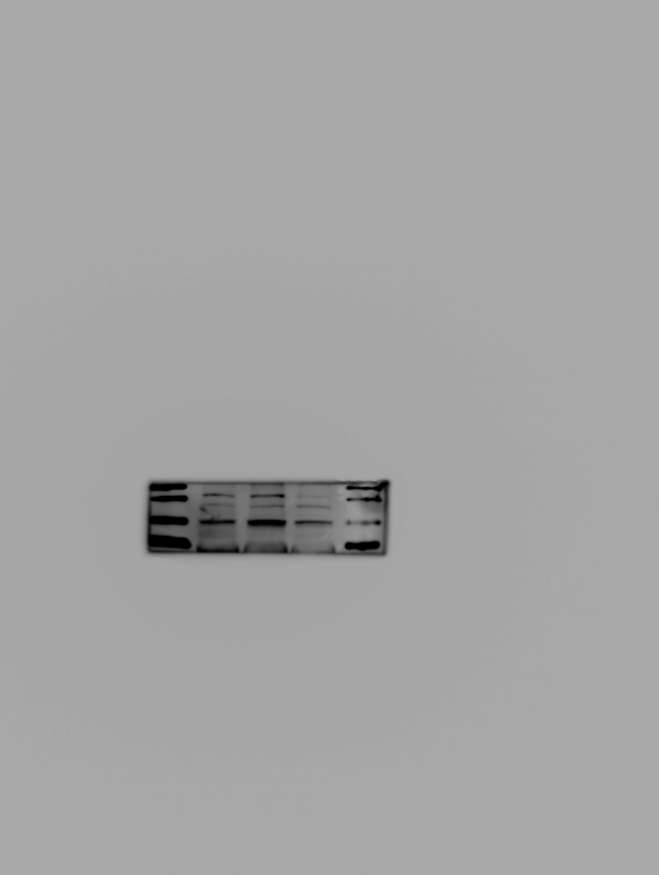


**Figure5D actin**


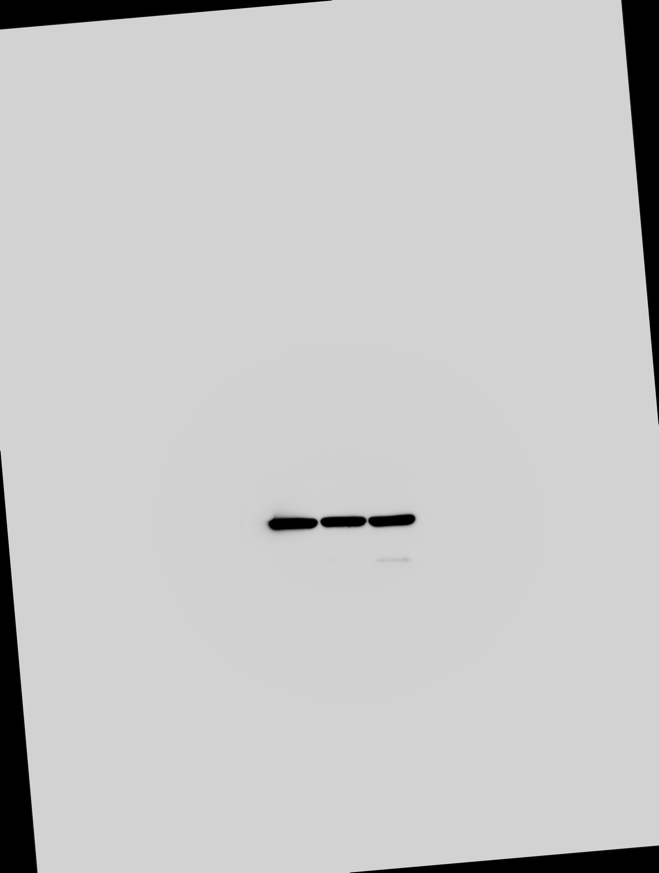


**Figure5D-drp1**


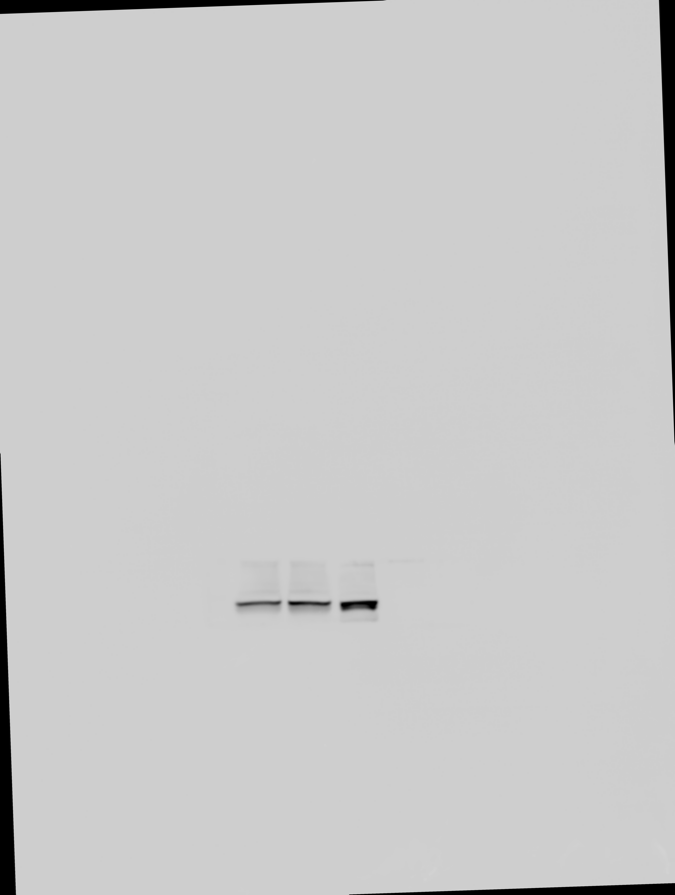


**Figure5D p-drp1**


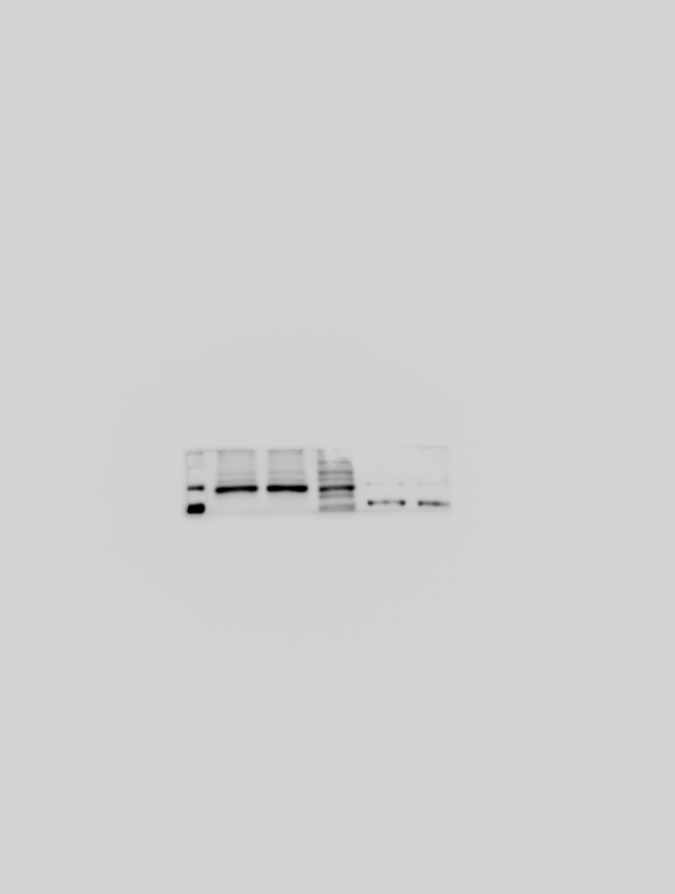


**Figure6A IB STX17**


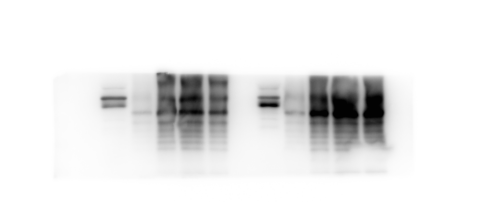


**Figure6A IB DRP1**


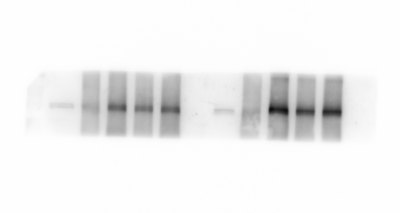


**Figure6G STX17**


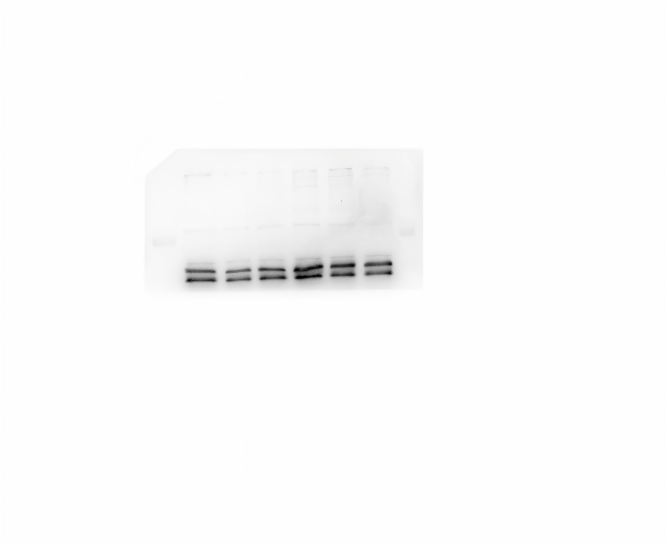


**Figure6G TOM20**


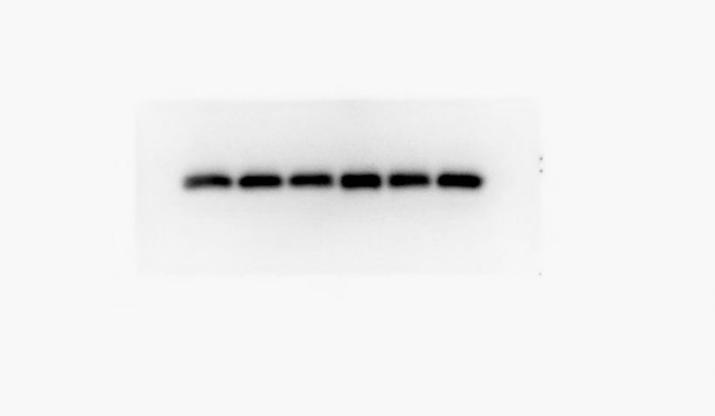


**Figure6G β-ACTIN**


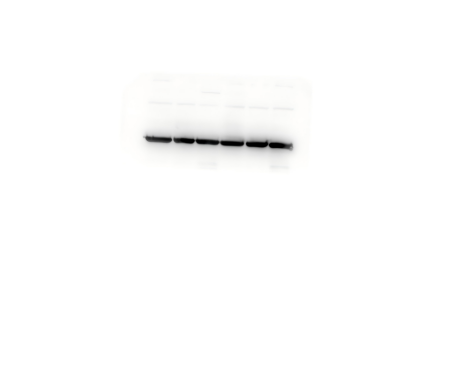


**FigureS3A DRP1 pSer616**


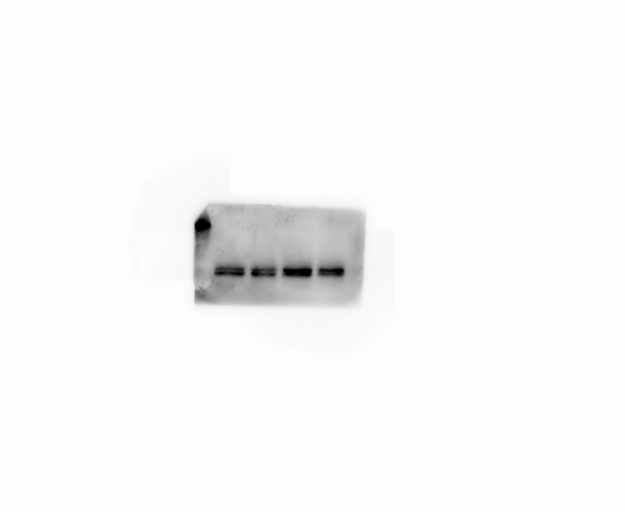


**FigureS3A DRP1**


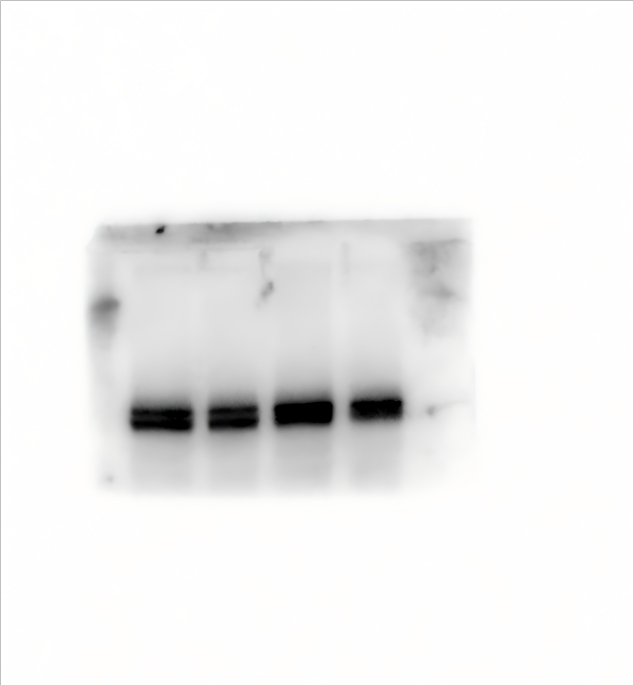


**FigureS3A Actin**


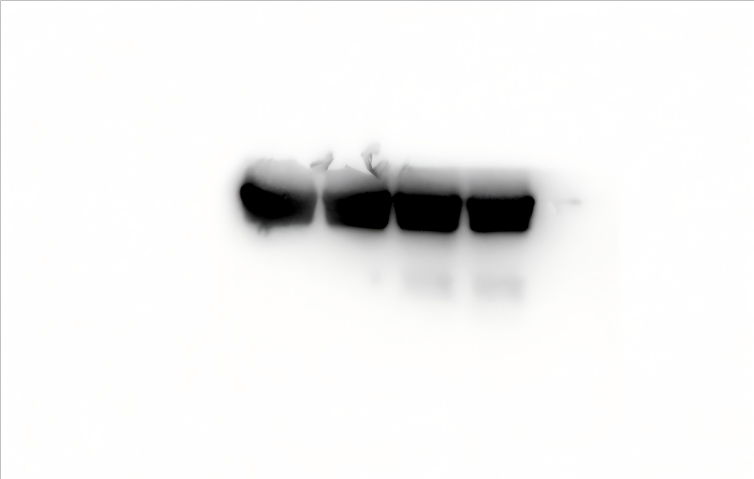


**FigureS4B pSer 1292**


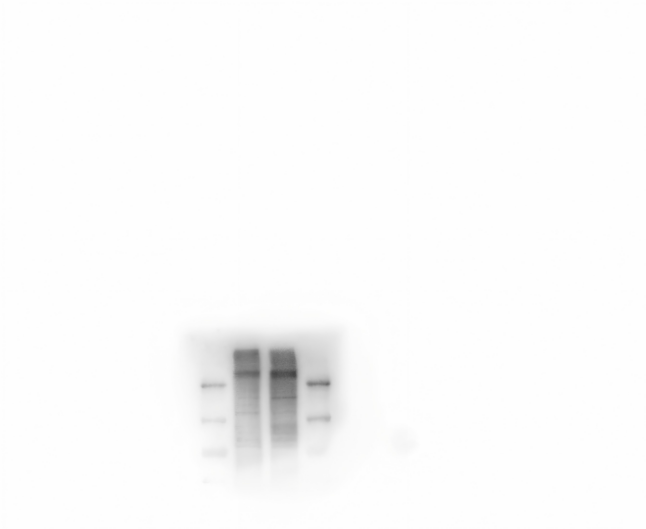


**FigureS4B LRRK2**


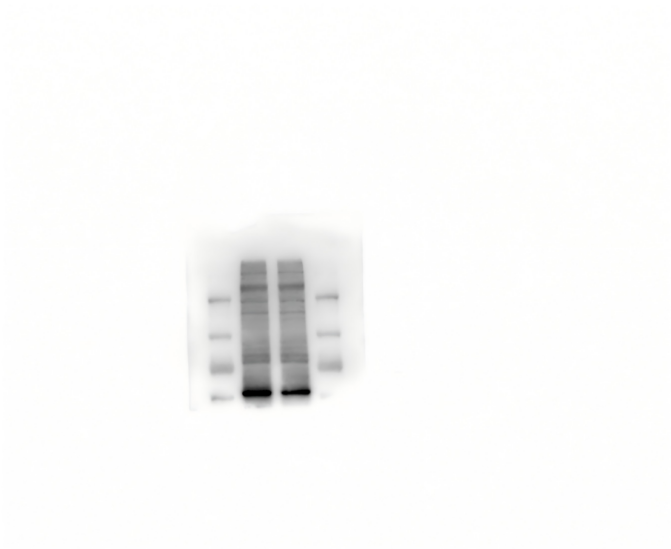


**FigureS4B β-actin**


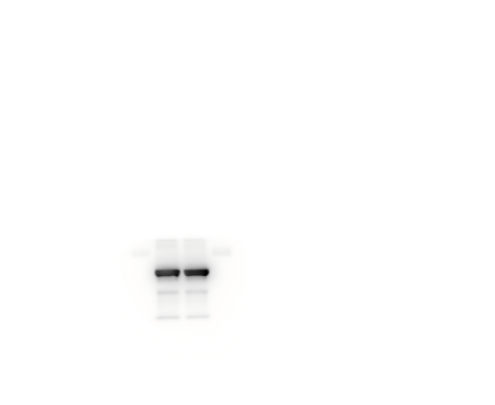


**FigureS5A β-actin**


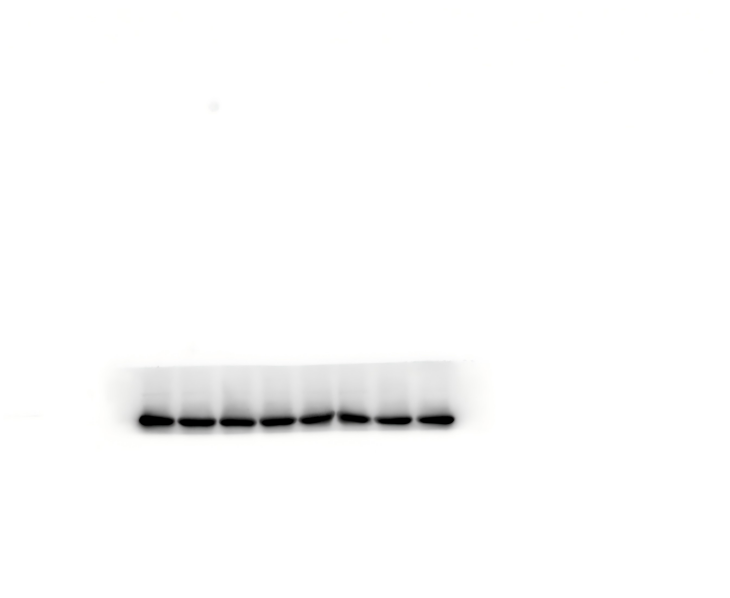


**FigureS5A PARKIN**


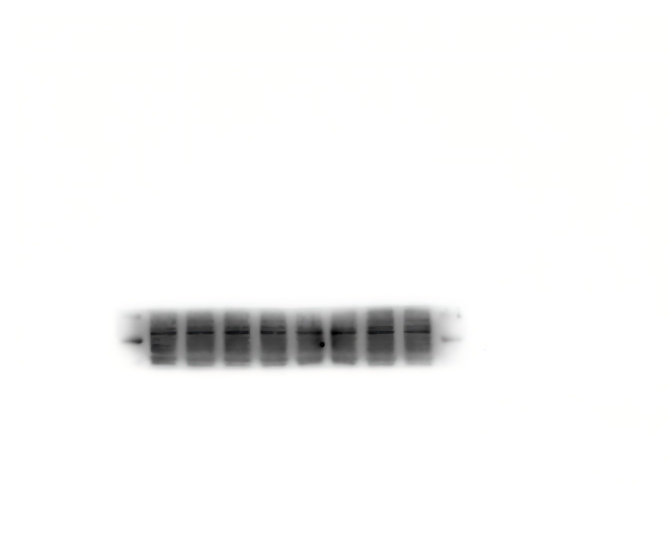


**FigureS5A Pink**


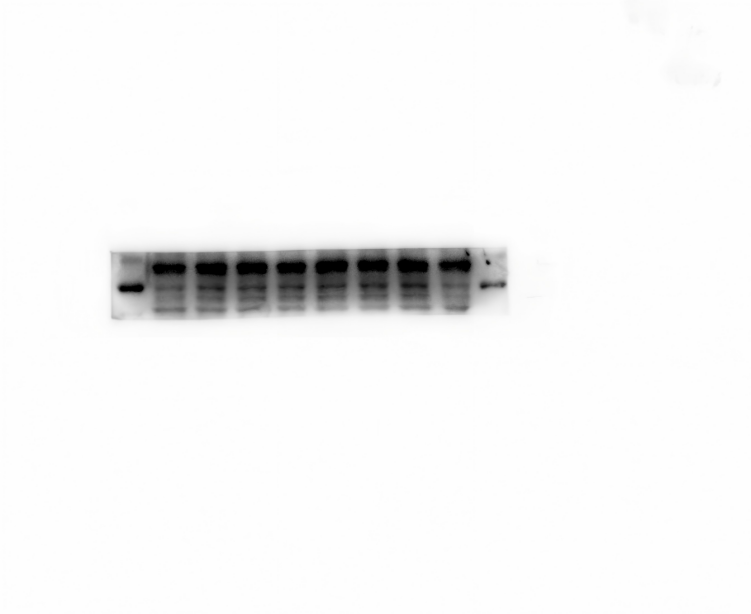


**FigureS5A pink pTHr257**


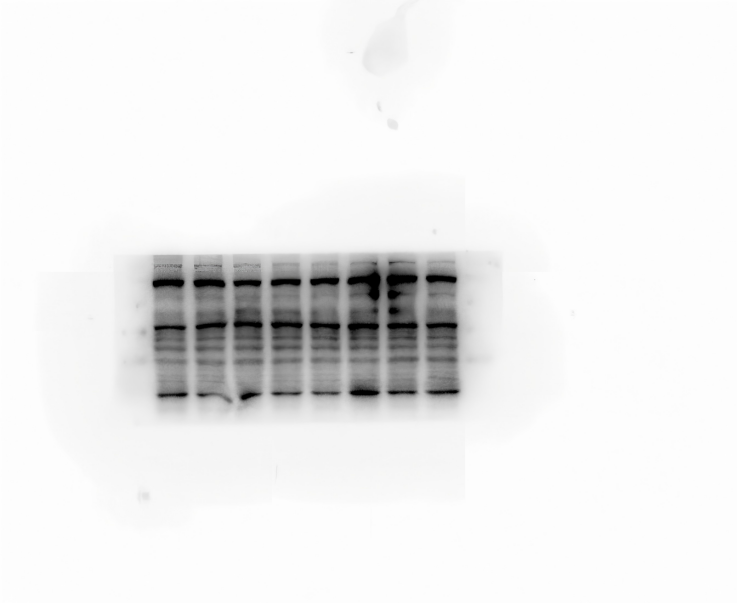


**FigureS5A PARKIN pSer65**


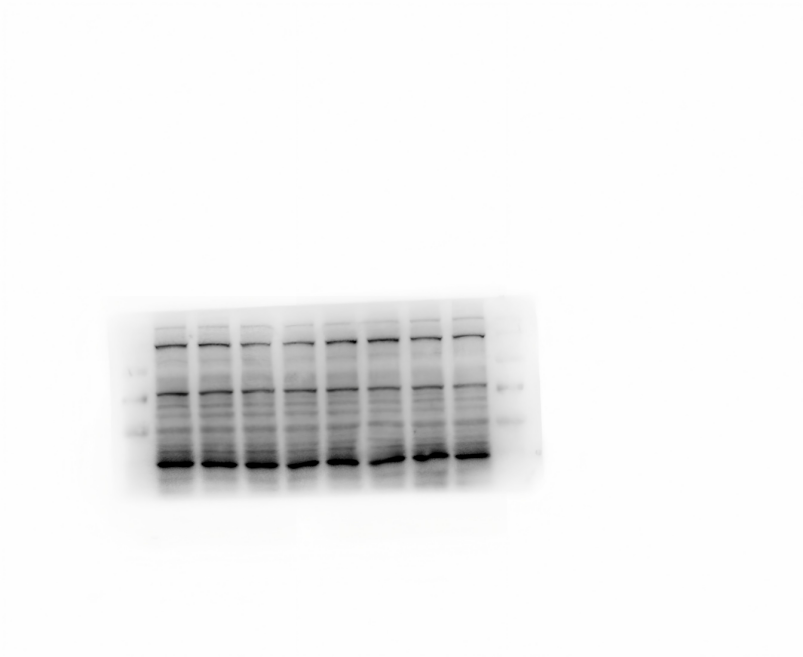

Supplement: Supplementary file 2 — Additional file 2. All original, full-length gel and blot images. [file 40035_2025_525_MOESM2_ESM.docx]
